# Systematic literature review and meta-analysis of clinical outcomes and prognostic factors for melanoma brain metastases

**DOI:** 10.3389/fonc.2022.1025664

**Published:** 2022-12-08

**Authors:** Xiang-Lin Tan, Amy Le, Emilie Scherrer, Huilin Tang, Nick Kiehl, Jiali Han, Ruixuan Jiang, Scott J. Diede, Irene M. Shui

**Affiliations:** ^1^ Merck & Co., Inc., Rahway, NJ, United States; ^2^ Department of Epidemiology, Richard M. Fairbanks School of Public Health, Indiana University, Indianapolis, IN, United States; ^3^ Seagen Inc., Bothell, WA, United States; ^4^ Integrative Precision Health, LLC, Carmel, IN, United States

**Keywords:** melanoma, brain metastasis, immunotherapy, targeted therapy, radiosurgery, prognostic factors, outcomes

## Abstract

**Background:**

More than 60% of all stage IV melanoma patients develop brain metastases, while melanoma brain metastases (MBM) is historically difficult to treat with poor prognosis.

**Objectives:**

To summarize clinical outcomes and prognostic factors in MBM patients.

**Methods:**

A systematic review with meta-analysis was conducted, and a literature search for relevant studies was performed on November 1, 2020. Weighted average of median overall survival (OS) was calculated by treatments. The random-effects model in conducting meta-analyses was applied.

**Results:**

A total of 41 observational studies and 12 clinical trials with our clinical outcomes of interest, and 31 observational studies addressing prognostic factors were selected. The most common treatments for MBM were immunotherapy (IO), MAP kinase inhibitor (MAPKi), stereotactic radiosurgery (SRS), SRS+MAPKi, and SRS+IO, with median OS from treatment start of 7.2, 8.6, 7.3, 7.3, and 14.1 months, respectively. Improved OS was observed for IO and SRS with the addition of IO and/or MAPKi, compared to no IO and SRS alone, respectively. Several prognostic factors were found to be significantly associated with OS in MBM.

**Conclusion:**

This study summarizes pertinent information regarding clinical outcomes and the association between patient characteristics and MBM prognosis.

## Introduction

Brain metastasis is a frequent and grave complication of melanoma (1). The median overall survival (OS) of patients with melanoma brain metastases (MBM) has historically been approximately 4 months after diagnosis. Recent studies have shown that immune checkpoint inhibitors targeting the programmed cell death protein 1 (PD1) and cytotoxic T-lymphocyte associated protein 4 (CTLA-4) pathways as well as novel small-molecule tyrosine kinase inhibitors targeting BRAF driver mutations, can improve survival in MBM (2). Margolin et al. reported a phase II trial investigating the activity of ipilimumab in MBM patients and showed that it was safe and resulted in tumor regression in some patients (3). Long et al. studied dabrafenib in BRAF mutated MBM in a phase II clinical trial, and demonstrated activity against brain metastases in MBM patients with or without prior local treatment (4). The treatment of MBM has thus shifted significantly in recent years, creating a growing body of research on novel targeted therapies in MBM in the realm of clinical oncology. However, there is still a lack of understanding of the efficacy of newer therapies for patients with MBM.

It has been suggested that patients who present with larger, symptomatic metastases are at higher risk for poorer performance status and worse prognosis, providing a strong rationale for early detection and treatment of MBM (5). An institutional database study of patients with melanoma enrolled on clinical trials from 1986 to 2004 by Davies et al. found that 330 developed MBM and prognostic factors for OS were earlier diagnosis, increased number of MBM, leptomeningeal involvement, and development of MBM after systemic therapy for extracranial metastatic disease (6). Nevertheless, prognostic factors for OS in MBM patients are not well defined.

To address these gaps in the research literature, there is a need to summarize the clinical outcomes and prognostic factors in patients with MBM at diagnosis or who develop MBM during the course of treatment. We performed a systematic review and meta-analysis to examine clinical outcomes, including OS and progression-free survival (PFS), and prognostic factors for patients with MBM, focusing on the most recent research.

## Patients and methods

### Study design and search strategy

This study was performed in accordance with the Preferred Reporting Items for Systematic Reviews and Meta-Analysis (PRISMA) guidelines. Relevant studies with full text articles published in English in the last five years were searched in the databases: EMBASE, MEDLINE, Cochrane Register of Controlled Trials, and Cochrane Review on November 1, 2020. Search terms included “melanoma”, “brain metastasis” or “cerebral metastasis”, and related terms (e.g. metastases), along with an epidemiology studies filter to include the eligible study designs (**Tables S1–S4**). Eligible studies were identified and selected according to the following eligibility criteria: 1) Studies published from November 1, 2015 to November 1, 2020; 2) study population are adult patients (>18 years) with melanoma who develop or present with at least one brain metastases; 3) reported clinical outcomes (OS, PFS) or prognostic factors for OS in MBM patients; 4) study designs included prospective and retrospective cohort studies, case-controls, cross-sectional studies, controlled or uncontrolled longitudinal studies; 5) no minimum sample sizes were required. Exclusion criteria included that the study was not published in English language, that the study was in animals or laboratory setting only, did not fall within the date range (published before November 1, 2015), had a duplicate study population, or if the relevant intervention (treatment) or outcomes of interest (OS, PFS, HR) were not available. Two reviewers independently selected studies according to the inclusion and exclusion criteria and extracted data, with a third independent reviewer available to address any discrepancies and perform a quality check. Bibliographies from review articles were reviewed thoroughly to identify relevant additional studies and trial results.

### Data extraction

The clinical outcomes of interest for this study were OS and PFS. We extracted median OS/PFS (in months) and the hazard ratios (HR) for OS/PFS along with 95% confidence intervals (CI). Some studies reported OS/PFS from date of diagnosis of MBM (time between diagnosis of brain metastases and death or last follow up), while others reported OS/PFS from start of treatment (time from the first treatment start date to the time of death or last follow-up). We also extracted the HR and 95% CI for each prognostic factor for OS in MBM patients, including age, sex, biomarkers, performance status, intracranial and extracranial disease status, and mutation status.

### Data analysis

The weighted average (by sample size) was calculated for the median months of OS by treatments. For studies that presented Kaplan-Meier (K-M) survival data without reporting HR, we used a previously published methodology for estimating HR from time-to-event analyses (7). Meta-HR for OS with corresponding 95% CIs were calculated for clinical outcomes and prognostic factors using random-effects models. Cochrane’s Q test and the I^2^ statistic were used to assess heterogeneity between studies, with a *P*-value < 0.05 for Cochrane’s Q test and I^2^ > 50% considered cut-offs for significant heterogeneity (8, 9). The results from the meta-analysis are presented graphically as forest plots. Publication bias was assessed by contour‐enhanced funnel plots of standard error against the effect estimate. All statistical analyses were performed using STATA (Version 14; Stata Corp., College Station, TX).

For clinical outcomes of observational studies, multiple studies were reported with clear information on treatment assignments for stereotactic radiosurgery (SRS) alone, MAP kinase inhibitor (MAPKi, which includes BRAFi [BRAF inhibitor] and/or MEKi [MEK inhibitor] and is used in patients with a BRAF mutation), SRS+IO, SRS+MAPKi, and SRS+MAPKi+IO. Therefore, we grouped those studies together, and performed meta-analyses for treatment comparisons by separating for those with OS from start of treatment and those with OS from date of diagnosis. However, if one study reported separate results for anti-PD1 and anti-CTLA-4 using a common reference group, these results were not grouped into a single IO group, but instead were reported separately in the summary tables.

For prognostic factors of MBM, the studies with similar definitions were grouped and meta-analysis was performed to summarize their association with OS in MBM patients. However, due to variable cut-off values and different reference groups chosen in some studies, we were not able to perform meta-analysis on all studies.

## Results

### Study selection

**Figure 1:**
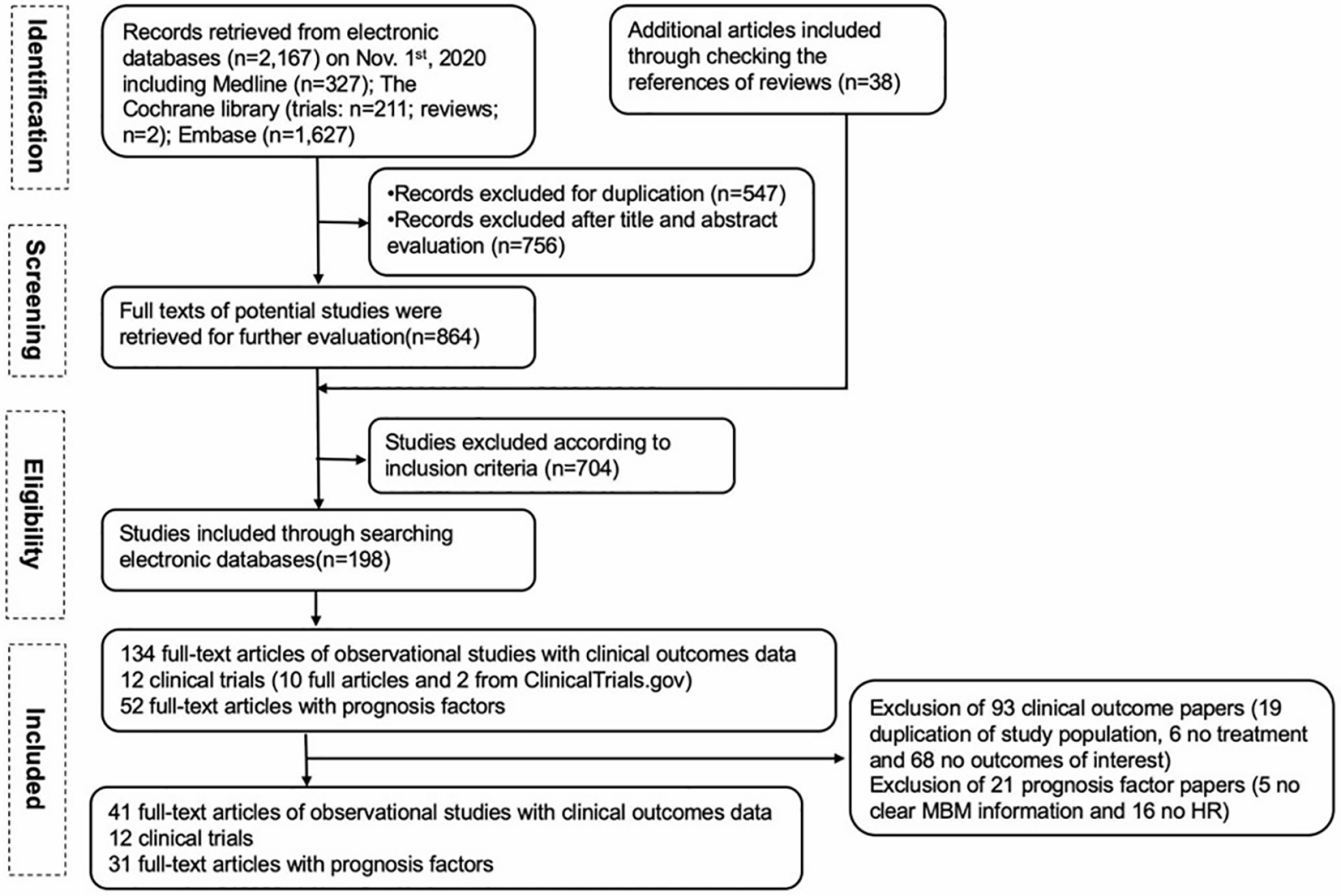
The study flow chart.

Our PRISMA study protocol is presented schematically in **Figure 1**. For clinical outcomes, 134 full-text articles of observational studies were screened, and 93 articles were excluded (19 due to duplication of the same population, 6 had no treatment reported, and 68 had no outcomes of interest). Ten full-text articles of clinical trials were included, and two additional clinical trials were identified from ClinicalTrials.gov. Finally, 41 observational studies and 12 clinical trials with our clinical outcomes of interest (OS and/or PFS) were included. For prognostic factors among MBM, 52 full-text articles were screened, and 21 were excluded (5 due to no clear MBM information, and 16 due to no HR). Thirty-one full-text papers for prognostic factors were included in the final analysis.

### OS reported in observational studies

We present the clinical outcomes from 41 observational studies in which median OS or HR for OS were available to extract, ordered either from start of treatment (29 studies) or from date of diagnosis (12 studies) (Supporting Information, **Tables S5, S6**) (10–50). The median OS averaged across studies utilizing the same treatment combinations is also shown in **Table 1**, ranging from 7.2-14.8 months from start of treatment and 6.2-16.6 months from date of diagnosis. For SRS+IO, the weighted average median OS was 14.1 months from start of treatment, and 16.6 months from date of diagnosis.

**Table 1:** Weighted average median overall survival (OS) in months by treatment.

Treatment	OS from Start of Treatment		OS from Data of Diagnosis	
	Number of studies	Median OS	Number of studies	Median OS
IO	4	7.2	4	14.6
MAPKi	3	8.6	2	13.7
SRS+ IO + MAPKi	1	14.8	0	N/A
SRS + IO	5	14.1	4	16.6
SRS + MAPKi	1	7.3	1	7.0
SRS	5	7.3	2	11.6
WBRT	0	N/A	4	6.2

IO, immunotherapy; MAPKi, MAP kinase inhibitor; OS, Overall survival; SRS, stereotactic radiosurgery; WBRT, whole brain radiation therapy.

### Meta-analysis by treatment for OS in observational studies

Meta-analysis by treatment for OS were summarized in **Table 2**, and forest plots were provided in **Figures S1-S6**. The significant benefit of IO on OS from start of treatment was observed by the comparison of SRS+IO *vs.* SRS alone (n = 8), with meta-HR of 0.48 (95% CI, 0.32-0.73). SRS compared to whole brain radiation therapy (WBRT) had a meta-HR of 0.55 (95% CI, 0.31-0.98) based on analysis of 2 studies (19, 20). Non-significant improvement of OS was observed for SRS+IO+MAPKi *vs.* SRS alone (meta-HR 0.40; 95% CI, 0.05-3.63; n=2), MAPKi *vs.* no MAPKi (meta-HR, 0.82; 95% CI, 0.46-1.46; n=3), and SRS+MAPKi *vs.* SRS alone (meta-HR, 0.71; 95% CI, 0.35-1.44; n=5) (11–13, 15, 16, 20–22).

**Table 2:** Meta-analysis by treatment for overall survival (OS) in observational studies.

Treatment	Reference		Number of studies	Meta-HR (95% CI)
*OS from Start of Treatment*				
MAPKi	No MAPKi		3	0.82 (0.46-1.46)
SRS + IO	SRS alone		8	0.48 (0.32-0.73)
SRS + MAPKi	SRS alone		5	0.71 (0.35-1.44)
SRS + IO + MAPKi	SRS alone		2	0.40 (0.05-3.63)
SRS	WBRT		2	0.55 (0.31-0.98)
*OS from Date of Diagnosis*				
IO	No IO		4	0.62 (0.45-0.86)
MAPKi	No MAPKi		2	0.45 (0.28-0.73)
SRS + IO	SRS alone		3	0.34 (0.15-0.81)
SRS + MAPKi	SRS alone		3	0.58 (0.33-1.03)
SRS	WBRT		3	0.78 (0.37-1.65)

IO, immunotherapy; MAPKi, MAP kinase inhibitor; Meta-HR, Meta-analysis hazard ratio; OS, Overall survival; SRS, stereotactic radiosurgery; WBRT, whole brain radiation therapy.

Meta-analysis results by treatment for OS from date of diagnosis showed similar results. For the OS from date of diagnosis, treatment with SRS+IO *vs.* SRS alone had meta-HR of 0.34 (95% CI, 0.15-0.81; n=3) (**Table 2**, **Figure S2**), and IO alone *vs.* no IO had a meta-HR of 0.62 (95% CI, 0.45-0.86; n=4) (**Table 2**, **Figure S6**) (39, 41, 42). For MAPKi vs. no MAPKi, meta-analysis showed meta-HR of 0.45 (95% CI, 0.28-0.73; n=2) (43, 50). However, no significant improvement OS from date of diagnosis was observed for SRS *vs.* WBRT or for SRS+MAPKi vs. SRS alone (39–42, 50).

### PFS reported in observational studies

Ten selected observational studies contained data on PFS, which ranged from 2-20.3 months from start of treatment or from 3.4-19 months from date of diagnosis (**Table S7**). Of the 10 studies, 9 also contained OS data, while one study by Robin et al. only included PFS data (51). PFS results were generally consistent with OS results, for example the study by Minniti et. al., 2019 that showed improved OS with SRS+IO found median PFS of 19 months from date of diagnosis of MBM (31).

### OS reported in clinical trials

The median OS and HR for OS results in 12 identified clinical trials are summarized in **Table S8** (52–63). Eleven clinical trials reported median OS ranging from 2.5 months (in patients who received only WBRT) to OS not reached (NR) in patients who received IO. However, comparison between trials was difficult given the different interventions being tested, the different patient populations (e.g. symptomatic vs. asymptomatic, previously treated vs. untreated, etc), and the relatively small numbers of patients in most trials (8 of the 12 trials had 25 patients or less in a study arm).

### Prognostic factors for OS in patients with MBM

The HRs for each prognostic factor extracted from 31 observational studies are summarized in **Table S9**, meta-HR are summarized in **Table 3**, and forest plots provided in **Figures S7–S15** (1, 27, 47, 48, 50, 64–89). Meta-analysis suggested elevated lactate dehydrogenase (LDH) levels, male gender, BRAF wild-type, increased number of intracranial metastases, presence of active extracranial metastases, lower Karnofsky Performance Scale (KPS), and larger MBM volume were significantly associated with worse prognosis in patients with MBM.

**Table 3:** Meta-analysis hazard ratios (Meta-HR) for prognostic factors of overall survival (OS) among patients with MBM.

Prognostic Factor	Comparison Group	Number of studies	Meta-HR (95% CI)
LDH	High *vs.* Normal	5	1.66 (1.19-2.30)
Sex	Male *vs.* Female	5	1.38 (1.10-1.74)
BRAF	Mutated *vs.* Wild-type	9	0.66 (0.52-0.83)
Intracranial metastases	≥ 4/5 MBM *vs.* 1 MBM	6	2.27 (2.08-2.48)
Extracranial metastases	Active *vs.* Controlled	8	1.86 (1.35-2.56)
KPS	≤70 *vs.* >70	4	2.73 (1.72-4.33)
	≤70 *vs.* >90-100	2	2.70 (1.80-4.06)
	> 80 *vs.* ≤80	2	4.23 (1.28-13.95)
	≥90 *vs.* <90	2	3.18 (2.02-5.00)
Brain metastases volume	Larger *vs.* smaller	2	1.02 (1.01-1.03)
Leptomeningeal disease	Present *vs.* Absent	2	2.36 (0.99-5.62)
Age	Continuous	7	1.01 (1.00-1.02)
	≥ 65 *vs.* <65 years	2	1.07 (0.72-1.57)

KPS, Karnofsky Performance Scale; LDH, lactate dehydrogenase; MBM, melanoma brain metastases; Meta-HR, Meta-analysis hazard ratio.

In particular, five studies showed increased LDH was associated with shorter survival (meta-HR, 1.66; 95% CI, 1.19-2.30). Five studies tested for an association between gender and OS and found decreased OS with male gender compared to female gender (meta-HR, 1.38; 95% CI, 1.10-1.74; n=5). Nine studies showed improved outcomes with BRAF mutation compared to BRAF wildtype (meta-HR, 0.66; 95% CI, 0.52-0.83; n=9). Nine studies assessed whether higher burden of MBM was associated with OS. In general, the data supported that more abundant intracranial metastases are associated with decreased OS. Among studies that had a reference group of 1 MBM compared to higher numbers, patients with 2 to 4 or 5 metastases had a meta-HR of 1.41 (95% CI, 1.11-1.80; n=5), and patients with more than 4 or 5 metastases had a meta-HR of 2.27 (95% CI, 2.08-2.48; n=6). Eight studies demonstrated worse survival outcomes with active extracranial disease compared to controlled disease (meta-HR, 1.86; 95% CI, 1.35-2.56). Decreased KPS (worse performance status) was associated with worse prognosis based on the results of thirteen studies, and the meta-HR was 2.73 (95% CI, 1.72-4.33; n=4), 4.23 (95% CI, 1.28-13.95; n=2), or 3.18 (95% CI, 2.02-5.00; n=2), using (≤70 *vs.* >70), (≤80 *vs.* >80), or (≤90 *vs.* >90) as cutoff points, respectively. Compared to those with KPS 90-100, those with KPS of ≤ 70 had a meta-HR of 2.70 (95% CI, 1.80-4.06; n=2). Larger total intracranial tumor volume was found to be associated with worse survival (meta-HR = 1.02; 95% CI, 1.01-1.03; n=2). Presence of leptomeningeal disease and advanced age trended towards association with worse prognosis, however the meta-HRs were non-significant.

## Discussion

Overall, evidence from observational studies suggest that SRS with addition of IO or IO plus MAPKi may improve survival outcomes in patients with MBM, compared to SRS alone. When averaged across studies utilizing the same treatment combinations, SRS+ IO had an improved median OS in months from start of treatment of approximately 14.1 months based on 5 studies. Treatment with combined SRS+IO+MAPKi was also promising with one study showing a median OS of 14.8 months. Meta-analyses provided support for the benefit from SRS+IO compared to SRS alone (12, 15). Further meta-analysis for studies that measured OS from date of diagnosis also showed that IO and SRS+IO had significantly improved OS compared to no IO and SRS alone, respectively.

A recent meta-analysis of MBM patients by Tawbi et al. (90) included 13 trials, of which 3 were randomized controlled trials, 9 were single-arm studies, and 1 was a non-randomized comparative study. They calculated median OS through a meta-analysis of K-M curves for selected interventions including IO or TT or as a weighted average of median OS. They observed that median OS was longer with nivolumab plus ipilimumab (28.3 months; 95% CI = 19.7-31.9) than with the other interventions including IO monotherapy or TT (range 5.7-11.8 months), based on pooled K-M curves. Similar OS benefit was also observed with nivolumab plus ipilimumab when the weighted average of the median was used (median OS 29.2 months) compared with the other interventions. This analysis suggested a clinical advantage with this treatment combination, but the heterogeneity of the data with respect to prior therapies (many patients received prior surgery, RT, systemic therapy, IO, or TT) and patient characteristics contributed uncertainty to the analysis.

Studies included in both the Tawbi et al. meta-analysis and our systematic review were a randomized trial by Long et al., 2018 and single-arm studies by McArthur et al., 2017, Davies et al., 2017, Kluger et al., 2019, and Tawbi et al., 2018 (52, 58–60, 62). However, in our analysis, prolonged median OS with IO was not demonstrated to the extent seen in the meta-analysis by Tawbi et al. In our study, average median OS from start of treatment was 7.2 months, 14.1 months, and 14.8 months for IO alone, SRS+IO, and SRS+IO+MAPKi, respectively. This may have been due to the heterogeneity of study populations, with inclusion of patients in the observational studies who had received a variety of prior treatments. Selection bias is also a limitation as healthier patients may be more likely to be selected for combination therapy such as SRS+IO or nivolumab+ipilimumab, and patients that undergo SRS generally have a limited number of brain metastases compared to patients that undergo WBRT or are not recommended for any radiation. It is worth noting that there may be unaccounted-for differences in patients who participated in clinical trials compared to those who did not (91). Given the variable patient populations and interventions, meta-analysis was not performed on the 12 clinical trials identified in our systematic review. More clinical trial data is needed for MBM patients in order to determine the most beneficial interventions.

In addition, our results suggest that elevated LDH levels, male gender, BRAF wild-type, more-numerous intracranial metastases, larger total MBM volume, presence of active extracranial metastases, and lower KPS scores may be prognostic for reduced OS in patients with MBM. While it is not unexpected that worse performance status and higher burden of disease were associated with reduced OS, some of the other associations are less clear. It is possible that an unknown confounding factor or biomarker is related to the association between gender and reduced OS. Limitations for this analysis included heterogeneity in participants, interventions, and outcomes studied (variable definitions in some studies related to the cutoff values and reference groups for some prognostic factors). A limitation of the OS meta-analysis results is that many studies defined date of diagnosis as the start date for OS calculation, rather than defining the start date as the day of treatment start, leading to more variability. Overall, this population is difficult to study given most of the data available is from retrospective reports or small clinical trials. Many of the meta-analyses performed included only a small number of studies. Since immunotherapy was not the primary focus, additional prognostic biomarkers such a neutrophil-lymphocyte ratio and PD-L1 were not included in this review. We have stayed abreast of the new literature on this specific topic after the date of our search execution. However, no major studies fell into our inclusion criteria.

In conclusion, although MBM is known to be associated with poor survival, evidence from our systematic review and meta-analysis of observational studies indicates that IO or combination IO and MAPKi therapy with SRS may lead to improved outcomes compared to patients treated without these therapies. A better understanding of prognostic factors may help clinicians with treatment planning, outcome assessment, and planning of support measures for individual MBM patients. Larger, randomized clinical trials would help to further elucidate the most effective therapy combinations to meet the needs of this understudied population.

## Data availability statement

The raw data supporting the conclusions of this article will be made available by the authors, without undue reservation.

## Author contributions

Study design, X-LT, ES, JH, and IS. Data collection, HT, NK, and JH. Data analysis, HT and JH. Manuscript writing, X-LT, AL, and JH. Manuscript review and approval, X-LT, AL, ES, HT, NK, JH, RJ, SD, and IS. All authors contributed to the article and approved the submitted version.
