# Supplementary material for: Systematic literature review and meta-analysis of clinical outcomes and prognostic factors for melanoma brain metastases
Source: Front Oncol. 2022 Dec 8;12:1025664. doi: 10.3389/fonc.2022.1025664 (PMC9773194; doi:10.3389/fonc.2022.1025664)
Supplement: Supplementary file 1 [file DataSheet_1.docx]

**Supporting Information**

**Table S1. Search strategy for EMBASE**

| No. | Criteria | Strings | Hits |
| --- | --- | --- | --- |
| 1 | Melanoma filter | 'melanoma'/exp | 210,302 |
| 2 | Melanoma filter | melanoma*:ti,ab | 162,234 |
| 3 | Melanoma filter | 1 OR 2 | 212,080 |
| 4 | Brain metastases filter | (Brain OR cerebral):ti,ab | 1,579,992 |
| 5 | Brain metastases filter | Metastasis/exp | 655,077 |
| 6 | Brain metastases filter | ‘metastas*’:ti,ab | 512,119 |
| 7 | Brain metastases filter | 5 OR 6 | 779,968 |
| 8 | Brain metastases filter | 4 AND 7 | 44,184 |
| 9 | Melanoma and brain metastases filter | 3 AND 8 | 5,598 |
| 10 | Epidemiology studies filter | ‘clinical study’ | 3,986,651 |
| 11 | Epidemiology studies filter | ‘case-control study’/exp | 173,377 |
| 12 | Epidemiology studies filter | ‘family study’ | 29,964 |
| 13 | Epidemiology studies filter | ‘longitudinal study’ | 161,729 |
| 14 | Epidemiology studies filter | ‘retrospective study’ | 968,293 |
| 15 | Epidemiology studies filter | ‘prospective study’ | 669,213 |
| 16 | Epidemiology studies filter | ‘randomized controlled trial’ | 801,128 |
| 17 | Epidemiology studies filter | ‘randomized controlled trial’/exp | 608,492 |
| 18 | Epidemiology studies filter | ‘clinical trial’ | 1,568,24 |
| 19 | Epidemiology studies filter | ‘Controlled clinical trial’ | 452,586 |
| 20 | Epidemiology studies filter | ‘cross-sectional study’ | 452,586 |
| 21 | Epidemiology studies filter | ‘cohort analysis’ | 590,147 |
| 22 | Epidemiology studies filter | ‘follow-up’ | 1,984,233 |
| 23 | Epidemiology studies filter | cohort*:ti,ab | 994,338 |
| 24 | Epidemiology studies filter | 22 and 23 | 259,890 |
| 25 | Epidemiology studies filter | 'case control':ti,ab | 156,198 |
| 26 | Epidemiology studies filter | (cohort NEAR/1 (study or studies or analys*)):ti,ab | 329,160 |
| 27 | Epidemiology studies filter | (('follow up' or observational or uncontrolled or 'non randomized' or nonrandomzed or epidemiologic*) NEAR/1 (study or studies)):ti,ab | 346,751 |
| 28 | Epidemiology studies filter | ((longitudinal or retrospective or prospective or ‘cross sectional’) and (study or studies or review or analys* or cohort*)):ti,ab | 2,152,724 |
| 29 | Epidemiology studies filter | OR/10-21,24-28 | 7,144,727 |
| 30 | Epidemiology studies filter | 'case report':ti,ab | 428,647 |
| 31 | Epidemiology studies filter | letter: it | 1,106,368 |
| 32 | Epidemiology studies filter | editorial: it | 650,422 |
| 33 | Epidemiology studies filter | animals/exp not [humans]/lim | 5,454,548 |
| 34 | Epidemiology studies filter | OR/30-33 | 7,591,090 |
| 35 | Epidemiology studies filter | 29 NOT 34 | 6,862,324 |
| 36 | melanoma filter, brain metastases filter and epidemiology studies filter | 9 and 35 | 2,739 |
| 37 | Limits | limit 36 to English language and 2015 to now | 1,497 |

**Table S2. Search strategy for MEDLINE**

| No. | Criteria | Strings | Hits |
| --- | --- | --- | --- |
| 1 | melanoma filter | Exp melanoma/ OR melanoma$.mp | 134,954 |
| 2 | melanoma filter | Melanoma*.ti,ab. | 116,072 |
| 3 | Melanoma filter | 1 OR 2 | 134,954 |
| 4 | Brain metastasis filter | (brain or cerebral).ti,ab. | 1,191,239 |
| 5 | Brain metastasis filter | Exp metastasis/ | 202,984 |
| 6 | Brain metastasis filter | metastas*.ti,ab. | 356,545 |
| 7 | Brain metastasis filter | 5 OR 6 | 461,060 |
| 8 | Brain metastasis filter | 4 AND 7 | 23,371 |
| 9 | Melanoma and brain metastases filter | 3 AND 8 | 2,659 |
| 10 | Epidemiology studies filter | Epidemiologic studies/ | 8,347 |
| 11 | Epidemiology studies filter | exp randomized controlled trials/ | 137,324 |
| 12 | Epidemiology studies filter | exp Randomized controlled trial/ | 509,671 |
| 13 | Epidemiology studies filter | Clinical Trial/ | 523,575 |
| 14 | Epidemiology studies filter | exp case control studies/ | 1,087,760 |
| 15 | Epidemiology studies filter | exp cohort studies/ | 2,006,522 |
| 16 | Epidemiology studies filter | Case control.tw. | 125,318 |
| 17 | Epidemiology studies filter | (cohort adj (study or studies)).tw. | 206,054 |
| 18 | Epidemiology studies filter | Cohort analy$.tw. | 8,043 |
| 19 | Epidemiology studies filter | (Follow up adj (study or studies)).tw. | 49,211 |
| 20 | Epidemiology studies filter | (observational adj (study or studies)).tw. | 106,827 |
| 21 | Epidemiology studies filter | Longitudinal.tw. | 244,864 |
| 22 | Epidemiology studies filter | Retrospective.tw. | 530,553 |
| 23 | Epidemiology studies filter | Cross sectional.tw. | 352,713 |
| 24 | Epidemiology studies filter | Cross-sectional studies/ | 331,033 |
| 25 | Epidemiology studies filter | or/10-24 | 3,709,536 |
| 26 | Epidemiology studies filter | case report.tw. | 311,300 |
| 27 | Epidemiology studies filter | letter.pt. | 1,086,997 |
| 28 | Epidemiology studies filter | editorial.pt. | 533,829 |
| 29 | Epidemiology studies filter | exp animals/ not humans.sh. | 4,713,859 |
| 30 | Epidemiology studies filter | or/26-29 | 6,586,535 |
| 31 | Epidemiology studies filter | 25 not 30 | 3,532,641 |
| 32 | Melanoma and brain metastases filter and epidemiology studies filter | 9 and 31 | 805 |

**Ovid MEDLINE(R) and Epub Ahead of Print, In-Process & Other Non-Indexed Citations, Daily and Versions(R) 1946 to Nov, 2020; Search executed: Nov 1, 2020**

**Table S3. Search strategy for Cochrane Register of Controlled Trials**

| No. | Criteria | Strings | Hits |
| --- | --- | --- | --- |
| 1 | Melanoma filter | (melanoma*):title abstract keyword | 5,192 |
| 2 | Brain metastasis filter | (brain OR cerebral): title abstract keyword | 69,541 |
| 3 | Brain metastasis filter | (metastas*): title abstract keyword | 23,625 |
| 4 | Brain metastasis filter | 2 AND 3 | 2,277 |
| 5 | Melanoma and brain metastasis filter | 1 AND 4 | 253 |
| 6 | Limits | Limit 5 to 2015 to current | 205 |

**Table S4. Search strategy for Cochrane reviews**

| No. | Criteria | Strings | Hits |
| --- | --- | --- | --- |
| 1 | Melanoma filter | (melanoma*): title abstract keyword | 33 |
| 2 | Brain metastasis filter | (brain OR cerebral): title abstract keyword | 960 |
| 3 | Brain metastasis filter | (metastas*): title abstract keyword | 172 |
| 4 | Brain metastasis filter | 2 AND 3 | 18 |
| 5 | Melanoma and brain metastasis filter | 1 AND 4 | 2 |
| 6 | Limits | Limit 5 to 2015 to current | 2 |

**Table S5. Summary of Overall Survival (OS) in Months in MBM Patients in the Identified Observational Studies**

| **First author, year** | **Treatment group**  **(Categorized for Meta-analysis)** | **Number of Patients^a^** | **Median OS**  **(range, 95% CI, or IQR)** | **HR (95% CI)** |
| --- | --- | --- | --- | --- |
| ***OS from Start of Treatment (N = 29)*** | | | | |
| Acharya, 2017 [1] | SRS alone (SRS alone) | 54 | NA | 1 |
|  | SRS+IO (SRS+IO) | 18 | NA | 0.53 (0.23-1.22) |
| An, 2017 [2] | SRS alone (SRS alone) | 99 | NA | 1 |
|  | SRS+IO (SRS+IO) | 23 | NA | 0.53 (0.21-1.30) |
|  | SRS+BRAFi (SRS+MAPKi) | 9 | NA | 0.68 (0.22-2.12) |
|  | SRS+Chemotherapy (NI) | 11 | NA | 1.35 (0.52-3.47) |
| Carron, 2020 [3] | SRS alone (SRS alone) | 18 | NA | 1 |
|  | SRS+BRAFi±MEKi (SRS+MAPKi) | 20 | NA | 1.85 (0.61-5.57) |
|  | Ipilimumab+SRS (SRS + IO) | 6 | NA | 0.48 (0.11-2.06) |
|  | SRS+BRAFi/MEKi+ ipilimumab (SRS+IO+MAPKi) | 5 | NA | 1.43 (0.26-7.78) |
| Choong, 2017 [4] | SRS alone (SRS alone) | 26 | NA | 1 |
|  | SRS+IO (SRS+IO) | 39 | NA | 0.51 (0.25-1.05) |
|  | SRS+BRAFi±MEKi (SRS+MAPKi) | 39 | NA | 0.3 (0.14-0.64) |
| Diao, 2018 [5] | SRS alone (SRS alone) | 40 | NA | 1 |
|  | SRS+concurrent ipilimumab (SRS+IO) | 23 | NA | 0.6 (0.32-1.11) |
|  | SRS+Non-concurrent ipilimumab (SRS+IO) | 28 | NA | 0.51 (0.28-0.92) |
| Gaudy-Marqueste, 2017 [6] | TT or IO before SRS -yes vs. no (NI) | 179 | NA | 0.99 (0.65-1.49) |
|  | TT or IO after SRS-yes vs. no (NI) | 179 | NA | 0.33 (0.22-0.49) |
|  | *BRAF mutants:* | | | |
|  | SRS alone (SRS alone) | 29 | 2.62 (NA) | 1 |
|  | SRS+BRAFi±MEKi (SRS+MAPKi) | 34 | 7.31 (NA) | 0.42 (0.22-0.80) |
|  | SRS+BRAFi±MEKi+IO (SRS+IO+MAPKi) | 31 | 14.82 (NA) | 0.15 (0.07-0.31) |
|  | SRS+IO (SRS+IO) | 11 | NA | 0.13 (0.04-0.39) |
|  | *BRAF wild-type:* | | | |
|  | SRS alone (NI) | 36 | 2.29 (NA) | 1 |
|  | SRS+anti-PD1 (NI) | 11 | 12.26 (NA) | 0.16 (0.05-0.48) |
|  | SRS+anti-CTLA-4 (NI) | 17 | 8.62 (NA) | 0.18 (0.07-0.44) |
|  | SRS+anti-CTLA-4+anti-PD1 (NI) | 4 | 14.07 (NA) | 0.28 (0.05-1.38) |
| Kaidar-Person, 2017 [7] | SRS alone (SRS alone) | 18 | 5.5 (3.8-8.4)^b^ | 1 |
|  | SRS+IO (SRS+IO) | 21 | 15 (10-15.8)^b^ | 0.34 (0.19-0.60) |
|  | SRS+BRAFi or MEKi (SRS+MAPKi) | 58 | NA | 1.47 (0.70-3.00) |
| Matsunaga, 2018 [8] | IO before SRS vs. SRS alone (SRS+IO vs. SRS alone) | 177 | NA | 1.18 (0.67-2.08) |
| Minniti, 2017 [9] | SRS alone (SRS alone) | 60 | 12 (NA) | 1 |
|  | SRS+Surgery (NI) | 60 | 14 (NA) | NA |
|  | SRS+IO (SRS+IO) | 120 | NA | 0.45 (0.27-0.83) |
| Rauschenberg, 2019 [10] | SRS vs. WBRT (SRS vs. WBRT) | 208 | NA | 0.4 (0.2-0.8) |
|  | TT vs. IO (NI) | 208 | NA | 3.2 (1.7-6.3) |
| Bhatia, 2019 [11] | Anti-CTLA-4 vs. anti-PD-1+anti-CTLA-4 (NI) | 77 | NA | 1.02 (0.50-2.07) |
|  | Anti-PD-1 vs. anti-PD-1+anti-CTLA-4 (NI) | 25 | NA | 0.49 (0.17-1.37) |
|  | SRS vs. WBRT (SRS vs. WBRT) | 69 | NA | 0.72 (0.42-1.23) |
|  | prior BRAFi±MEKi vs. prior chemotherapy (NI) | 42 | NA | 1.03 (0.44-2.32) |
|  | Prior BRAFi vs. no (MAPKi vs. no MAPKi) | 88 | NA | 1.63 (0.81-3.29) |
| Gorka, 2018 [12] | BRAFi after local control vs. local therapies/chemotherapy (MAPKi vs. no MAPKi) | 30 | 11.8 (NA) | 0.45 (0.27-0.74) |
| Iorgulescu, 2018 [13] | Checkpoint Blockade IO, yes vs. no  (IO vs. no IO) | 880 | NA | 0.42 (0.29-0.63) |
|  | BRAFi, yes vs. no  (MAPKi vs. no MAPKi) | 850 | NA | 0.85 (0.66-1.09) |
|  | Single-fraction SRS, yes vs. no (NI) | 845 | NA | 0.53 (0.39-0.73) |
|  | Hypofractionated SRT, yes vs. no (NI) | 845 | NA | 0.88 (0.60-1.28) |
|  | WBRT, yes vs. no (NI) | 845 | NA | 1.12 (0.86-1.46) |
|  | Other RT regimen, yes vs. no (NI) | 845 | NA | 0.79 (0.55-1.13) |
| Ahmed, 2016 [14] | SRS+Chemotherapy (NI) | 20 | NA | 1 |
|  | SRS+Anti-PD-1 (NI) | 21 | NA | 0.29 (0.14-0.63) |
|  | SRS+Anti-CTLA-4 (NI) | 25 | NA | 0.32 (0.16-0.67) |
|  | SRS+BRAFi/MEKi (NI) | 12 | NA | 0.42 (0.19-0.91) |
|  | SRS+BRAFi (NI) | 18 | NA | 0.56 (0.27-1.12) |
| De La Fuente, 2015 [15] | WBRT (NI) | 61 | 3 (0-24)^a^ | NA |
| Drago, 2019 [16] | BRAFi + MEKi (NI) | 65 | 9.5 (7.7-13.5)^b^ | NA |
| Frakes, 2015 [17] | WBRT before SRS: no vs. yes (NI) | 28 | NA | 0.11 (0.02-0.53) |
|  | Concurrent WBRT with SRS: no vs. yes (NI) | 28 | NA | 0.69 (0.27-2.13) |
|  | WBRT after SRS: no vs. yes (NI) | 28 | NA | 3.46 (1.10-11.96) |
| Geukes Foppen, 2018 [18] | vemurafenib (NI) | 85 | 5.7 (4.6-6.8)^b^ | 1 |
|  | dabrafenib (NI) | 31 | 8.8 (3.9-13.7)^b^ | 0.8 (0.4-1.4) |
|  | dabrafenib+trametinib (NI) | 30 | 11.2 (6.8-15.7)^b^ | 0.6 (0.3-1.1) |
| Le Rhun, 2020 [19] | IO alone (NI) | 10 | 6 (3-23)^c^ | NA |
|  | SRS without or with non-IO (NI) | 20 | 14 (10-42)^c^ | NA |
|  | SRS+IO (NI) | 32 | 11 (7-28)^c^ | NA |
| Knispel, 2020 [20] | No preceding RT (NI) | 86 | 4.6 (2.4-9.8)^b^ | 1 |
|  | Preceding RT (NI) | 137 | 6.4 (2.7-14.8)^b^ | 0.77 (0.53-1.13) |
| McHugh, 2020 [21] | No TT or IO (NI) | 110 | 6.5 (3.8-9.1)^b^ | NA |
|  | TT ± IO (NI) | 110 | 12.3 (9.5-15.1)^b^ | NA |
| Minniti, 2019 [22] | SRS and ipilimumab (NI) | 45 | 14.7 (NA) | 1 |
|  | SRS and nivolumab (NI) | 35 | 22 (NA) | 0.51 (0.28-0.81) |
| Tetu, 2019 [23] | non-combined RT (NI) | 169 | 6.9 (5.4-9.4)^b^ | 1 |
|  | Combined RT (RT delivered at or nearest to the first systemic therapy dose (± 30 days) (NI) | 93 | 16.8 (11.8-27.9)^b^ | 0.6 (0.4-0.8) |
| Pomeranz Krummel, 2020 [24] | IO prior to RT vs. RT prior to IO (NI) | 17 | NA | 3.54 (0.86-14.65) |
| Rahman, 2018 [25] | Concurrent IO with RT (within 30 days of RT) (NI) | 35 | 17.8 (NA) | NA |
|  | Non-concurrent IO and RT (NI) | 39 | 11.6 (NA) | NA |
| Schmidberger, 2018 [26] | Anti-CTLA-4 after RT (NI) | 41 | 11 (NA) | 1 |
|  | Anti-CTLA-4 before RT (NI) | 41 | 3 (NA) | 2.7 (p=0.01) |
| Skrepnik, 2017 [27] | SRS+ipilimumab (NI) | 25 | 35.8 (NA) | NA |
| Stera, 2019 [28] | SRS+BRAFi vs. SRS+anti-CTLA‐4 (NI) | 48 | NA | 2.25 (1.06-4.75) |
|  | before/concomitant to SRS vs. after SRS (NI) | 48 | NA | 0.31 (0.11-0.88) |
|  | PD1/Combined vs. anti-CTLA-4 (NI) | 45 | NA | 0.47 (0.24-0.93) |
| Yusuf, 2017 [29] | SRS alone (NI) | 33 | 7.1 (1-51.8)^a^ | NA |
|  | SRS+IO (NI) | 18 | 7.4 (0.9-26.4)^a^ | NA |
| ***OS from Date of Diagnosis (N = 12)*** | | | | |
| Amaral, 2019 [30] | Surgery/SRS (SRS alone) | 14 | 4 (0-2.1)^b^ | 1 |
|  | Surgery/SRS + chemotherapy (NI) | 7 | 11 (8.4-13.6)^b^ | 0.6 (0.22-1.63) |
|  | Surgery/SRS + IO (SRS + IO) | 36 | 25 (14.6-35.4)^b^ | 0.25 (0.11-0.56) |
|  | Surgery/SRS +BRAFi+MEKi (SRS+MAPKi) | 16 | 14 (12.1-15.9)^b^ | 0.4 (0.16-1.00) |
|  | No RT (NI) | 35 | 3 (1.7-4.2)^b^ | 1.34 (0.65-2.79) |
|  | WBRT ± systemic therapy (WBRT) | 55 | 5 (3.9-6.1)^b^ | 0.85 (0.40-1.82) |
| Gabani, 2018 [31] | RT+IO (NI) | 192 | 11.1 (8.9-13.4)^b^ | NA |
|  | RT (NI) | 912 | 6.2 (5.6-6.8)^b^ | NA |
|  | SRS alone (SRS alone) | 274 | 11.9 (9.8-14)^b^ | NA |
|  | SRS+IO (NI) | 89 | 17 (10.7-23.3)^b^ | NA |
|  | WBRT+IO vs. WBRT (IO vs. no IO) | 741 | NA | 0.54 (0.42-0.70) |
|  | SRS vs. WBRT (SRS vs. WBRT) | 912 | NA | 0.44 (0.37-0.53) |
|  | SRS+IO vs. WBRT (NI) | 727 | NA | 0.33 (0.24-0.44) |
| Kotecha, 2018 [32] | SRS alone  SRS alone |  |  | 1 |
|  | SRS+BRAFi (SRS+MAPKi) | 366 | NA | 0.48 (0.28-0.92) |
|  | SRS+anti-PD1/anti-CTLA-4 (SRS+IO) | 366 | NA | 0.67 (0.44-0.99) |
|  | SRS+Cytokine therapy (NI) | 366 | NA | 0.54 (0.28-0.92) |
| Martins, 2020 [33] | SRS alone (SRS alone) | 84 | NA | 1 |
|  | SRS+anti-PD1±anti-CTLA-4 (SRS+IO) | 18 | 24 (17-NR)^b^ | 0.19 (0.07-0.50) |
|  | SRS+Anti-CTLA-4 (NI) | 10 | 7.5 (3-NR)^b^ | 2.87 (1.15-7.17) |
|  | SRS+BRAFi ± MEKi (SRS+MAPKi) | 11 | 7 (6-NR)^b^ | 1.16 (0.46-2.91) |
| Sloot, 2018 [34] | No-BRAFi, No-anti-CTLA‐4 (No IO, No MAPKi) | 243 | NA | 1 |
|  | BRAFi/No-anti-CTLA‐4 (MAPKi) | 243 | NA | 0.59 (0.27-1.30) |
|  | Anti-CTLA‐4/No-BRAFi (IO) | 243 | NA | 0.44 (0.26-0.74) |
|  | Anti-CTLA‐4/BRAFi (NI) | 243 | NA | 0.18 (0.04-0.77) |
|  | SRS alone (SRS alone) | 118 | 10.8 (9.6-16.8)^b^ | 1 |
|  | No treatment/Observation (NI) | 13 | 2.4 (0-4.8)^b^ | 3.5 (1.5-8.2) |
|  | Surgery (NI) | 40 | 12 (7.2-22.8)^b^ | 1.35 (0.70-2.59) |
|  | WBRT (WBRT) | 58 | 7.2 (4.8-15.6)^b^ | 0.92 (0.51-1.67) |
| Wattson, 2015 [35] | No IO, No MAPKi (No IO, No MAPKi) | 256 | 7 (5.6-8.3)^b^ | 1 |
|  | MAPKi (MAPKi) | 199 | 14.1 (8.3-27.7)^b^ | 0.39 (0.21-0.70) |
|  | local therapy:SRS or resection (NI) | 74 | NA | 0.59 (0.38-0.90) |
|  | WBRT (NI) | 46 | NA | 1.3 (0.88-2.10) |
|  | IO (IO) | 8 | NA | 1.4 (0.84-2.60) |
|  | cytotoxic chemotherapy (NI) | 37 | NA | 1.09 (0.71-1.70) |
| Stokes, 2017 [36] | No IO (No IO) | 1102 | 6.1 (NA) | 1 |
|  | IO (IO) | 185 | 10.8 (NA) | 0.57 (0.47-0.70) |
|  | Chemotherapy vs. not (NI) | 1287 | NA | 0.70 (0.61-0.80) |
|  | SRS vs. conventionally-fractionated RT (NI) | 1052 | NA | 0.52 (0.46-0.60) |
| Tio, 2018 [37] | Systemic therapy with BRAFi (NI) | 92 | 13.6 (11.5-16)^b^ | 1 |
|  | Systemic therapy+IO (NI) | 75 | 11.1 (5.1-17.1)^b^ | 1.17 (0.80-1.70) |
|  | Best supportive care (NI) | 48 | 1.8 (1.2-2.3)^b^ | 1 |
|  | Systemic therapy (NI) | 198 | 5.4 (3.1-7.7)^b^ | 0.33 (0.10-1.06) |
|  | WBRT (NI) | 122 | 4.4 (2.4-6.3)^b^ | 0.72 (0.23-2.29) |
|  | Systemic therapy+WBRT (NI) | 45 | 5.2 (4.1-6.4)^b^ | 0.47 (0.18-1.23) |
|  | SRS/surgery±WBRT (NI) | 70 | 6.4 (5.4-7.5)^b^ | 0.26 (0.11-0.66) |
|  | Systemic therapy + SRS/surgery (NI) | 126 | 14.9 (10.7-19)^b^ | 0.22 (0.09-0.54) |
| Alvarez-Breckenridge, 2019 [38] | Brain metastasis diagnosis followed by surgery and subsequent IO (NI) | 49 | NA | 1 |
|  | Brain metastasis diagnosis followed by IO (NI) | 48 | NA | 1.72 (1.00-2.99) |
|  | Brain metastasis diagnosis followed by IO and subsequent surgery (NI) | 11 | NA | 0.75 (0.30-1.91) |
|  | IO followed by brain metastasis diagnosis (NI) | 34 | NA | 1.87 (1.00-3.48) |
| Amaral, 2020 [39] | nivolumab+ipilimumab+surgery/SRS (first line) (NI) | 250 | 17 (10.7-23.9)^b^ | NA |
|  | nivolumab+ipilimumab+surgery/SRS (NOT first line) (NI) | 130 | 21 (17.8-24.2)^b^ | NA |
| Cohen-Inbar, 2017 [40] | SRS after ipilimumab treatment (NI) | 14 | 6.4 (1.2-24.5)^a^ | NA |
|  | SRS before or during ipilimumab cycles (NI) | 32 | 13.8 (2.7-43.2)^a^ | NA |
| White, 2020 [41] | IO (NI) | 141 | 11.5 (8-14.5)^b^ | NA |
|  | RT+IO (NI) | 141 | 10.8 (7.8-14.8)^b^ | NA |
|  | SRS+IO (NI) | 69 | 19 (12.3-28.6)^b^ | NA |
|  | WBRT+IO (NI) | 72 | 7.7 (6.4-9.8)^b^ | NA |

**Abbreviations:** Anti-CTLA-4 = Anti-cytotoxic T-lymphocyte associated protein 4; Anti-PD-1 = Anti-programmed cell death protein 1; BRAFi = BRAF inhibitor; IO = immunotherapy; MAPKi = MAP kinase inhibitor; MEKi = MEK inhibitor; NI = not included in meta-analysis; NA = not reported; NR = not reached; RT = radiation therapy; SRS = stereotactic radiosurgery; SRT = stereotactic radiation therapy; TT = targeted therapy; WBRT = whole brain radiation therapy

*****The number of patients is the number in each row of treatment group. If there is one group vs. the other group, the number of patients is the total patients among the two groups.

^a^ Range provided in the study

^b^ 95% CI provided in the study

^c^ Interquartile range (IQR) provided in the study

**Table S6. Median Overall Survival (OS) in Months by Treatments**

| **Treatment**  **Category** | **First author, year** | **Treatment group** | **Number of patients** | **Median OS (range, 95% CI, or IQR)** |
| --- | --- | --- | --- | --- |
| SRS+IO | Minniti, 2019 [22] | SRS+anti-CTLA-4 | 45 | 14.7 (NA) |
|  |  | SRS+anti-PD-1 | 35 | 22 (NA) |
|  | Yusuf, 2017 [29] | SRS+IO | 18 | 7.4 (0.9-26.4)^a^ |
|  | Kaidar-Person, 2017 [7] | SRS+IO | 29 | 15 (10-15.8)^b^ |
|  | Le Rhun, 2020 [19] | SRS +IO | 32 | 11 (7-28)^c^ |
|  | Gaudy-Marqueste, 2017 [6] | SRS+anti-CTLA-4 | 17 | 8.6 (NA) |
|  |  | SRS+anti-CTLA-4+anti-PD-1 | 4 | 14.1 (NA) |
|  |  | SRS+anti-PD-1 | 11 | 12.3 (NA) |
| SRS alone | Le Rhun, 2020 [19] | SRS alone | 20 | 14 (10-42)^c^ |
|  | Minniti, 2017 [9] | SRS alone | 60 | 12 (NA) |
|  | Yusuf, 2017 [29] | SRS alone | 33 | 7.1 (1-51.8)^a^ |
|  | Kaidar-Person, 2017 [7] | SRS alone | 29 | 5.5 (3.8-8.4)^b^ |
|  | Gaudy-Marqueste, 2017 [6] | SRS alone | 70 | 2.3 (NA) |
| SRS+MAPKi + IO | Gaudy-Marqueste, 2017 [6] | SRS+BRAFi±MEKi+IO | 31 | 14.8 (NA) |
| SRS+MAPKi | Gaudy-Marqueste, 2017 [6] | SRS+BRAFi±MEKi | 34 | 7.3 (NA) |
|  | Drago, 2019 [16] | BRAFi+MEKi | 65 | 9.5 (7.7-13.5)^b^ |
|  | Gorka, 2018 [12] | BRAFi after local control | 30 | 11.8 (NA) |
|  | Geukes Foppen, 2018 [18] | BRAFi dabrafenib | 31 | 8.8 (3.9-13.7)^b^ |
|  |  | BRAFi vemurafenib | 85 | 5.7 (4.6-6.8)^b^ |
|  |  | BRAFi+MEKi dabrafenib+trametinib | 30 | 11.2 (6.8-15.7)^b^ |
| IO alone or in combination with RT | Knispel, 2020 [20] | IO, No preceding RT | 86 | 4.6 (2.4-9.8)^b^ |
|  |  | IO, Preceding RT | 137 | 6.4 (2.7-14.8)^b^ |
|  | Rahman, 2018 [25] | IO+RT | 35 | 17.8 (NA) |
|  | Schmidberger, 2018 [26] | anti-CTLA-4 after RT | 41 | 11 (NA) |
|  |  | anti-CTLA-4 before RT | 41 | 3 (NA) |
|  | Le Rhun, 2020 [19] | IO alone | 10 | 6 (3-23)^c^ |
| MAPKi | Gorka, 2018 [12] | BRAFi after local control vs. local therapies/ chemotherapy | 30 | 11.8 (NA) |
|  | Drago, 2019 [16] | BRAFi/MEKi | 65 | 9.5 (7.7-13.5)^b^ |
|  | Geukes Foppen, 2018 [18] | BRAFi dabrafenib | 31 | 8.8 (3.9-13.7)^b^ |
|  |  | BRAFi vemurafenib | 85 | 5.7 (4.6-6.8^)b^ |
|  |  | BRAFi+MEKi dabrafenib+trametinib | 30 | 11.2 (6.8-15.7)^b^ |
| ***OS from Date of Diagnosis*** | | | | |
| SRS+IO | Cohen-Inbar, 2017 [40] | SRS after ipilimumab cycles | 32 | 13.8 (2.7-43.2)^a^ |
|  |  | SRS before or during ipilimumab cycles | 14 | 6.4 (1.2-24.5)^a^ |
|  | Martins, 2020 [33] | SRS+anti-CTLA-4 | 10 | 7.5 (3-NR)^b^ |
|  |  | SRS+anti-PD-1±anti-CTLA-4 | 18 | 24 (17-NR) ^b^ |
|  | White, 2020 [41] | SRS+IO | 69 | 19 (12.3-28.6) ^b^ |
|  | Gabani, 2018 [31] | SRS+IO | 89 | 17(10.7-23.3) ^b^ |
| SRS alone | Sloot, 2018 [34] | SRS alone | 118 | 10.8 (9.6-16.8) ^b^ |
|  | Gabani, 2018 [31] | SRS alone | 274 | 11.9 (9.8-14) ^b^ |
| SRS+MAPKi | Martins, 2020 [33] | SRS+BRAFi±MEKi | 11 | 7 (6-NR) ^b^ |
|  | Tio, 2018 [37] | BRAFi | 92 | 13.6 (11.5-16) ^b^ |
|  | Wattson, 2015 [35] | MAPKi | 37 | 14.1 (8.3-27.7) ^b^ |
| IO alone or in combination with RT | Stokes, 2017 [36] | IO+RT | 185 | 10.8 (9.1-12.5) ^b^ |
|  | Amaral, 2020 [39] | IO not first line | 130 | 21 (17.8-24.2) ^b^ |
|  |  | IO+local therapy | 250 | 17 (10.7-23.9) ^b^ |
|  | White, 2020 [41] | IO | 142 | 11.5 (8-14.5) ^b^ |
|  | Tio, 2018 [42] | IO | 75 | 11.1 (5.1-17.1) ^b^ |
| MAPKi | Tio, 2018 [37] | BRAFi | 92 | 13.6 (11.5-16)^b^ |
|  | Wattson, 2015 [35] | MAPKi | 37 | 14.1 (8.3-27.7)^b^ |

**Abbreviations:** Anti-CTLA-4 = Anti-cytotoxic T-lymphocyte associated protein 4; Anti-PD-1 = Anti-programmed cell death protein 1; BRAFi = BRAF inhibitor; IO = immunotherapy; MAPKi = MAP kinase inhibitor; MEKi = MEK inhibitor; NA = not reported; NR = not reached; RT = radiation therapy; SRS = stereotactic radiosurgery; TT = targeted therapy

^a^ Range provided in the study

^b^ 95% CI provided in the study

^c^ IQR provided in the study

**Table S7. Summary of Progression Free Survival (PFS) in MBM Patients in the Identified Observational Studies (N= 10)**

| First author, year | Treatment group | Number of patients | Starting point of PFS^a^ | Median PFS in month (95% CI) | HR (95% CI) |
| --- | --- | --- | --- | --- | --- |
| Drago, 2019 [16] | BRAFi + MEKi | 65 | T | 5.3 (3.6-6.1) | NA |
| Geukes Foppen, 2018 [18] | vemurafenib (REF) | 85 | T | 3.6 (3.5-3.8) | 1 |
|  | dabrafenib | 31 | T | 5.7 (3-8.4) | 0.7 (0.4-1.2) |
|  | dabrafenib+trametinib | 30 | T | 5.8 (3.2-8.5) | 1 (0.6-1.6) |
| Knispel, 2020 [20] | IO with no preceding RT (REF) | 86 | T | 2.7 (1.4-3.5) | 1 |
|  | IO with Preceding RT | 137 | T | 2.8 (1.5-5.1) | 0.9 (0.6-1.2) |
| Minniti, 2019 [22] | SRS and ipilimumab (REF) | 45 | T | 6 (NA) | 1 |
|  | SRS and nivolumab | 35 | T | 10 (NA) | 0.5 (0.3-0.9) |
| Tetu, 2019 [23] | no-combined RT | 169 | T | 3.5 (2.7-4.5) | NA |
|  | combined RT | 93 | T | 5.2 (3.5-7.9) | NA |
| Robin, 2018 [43] | Anti-CTLA4 | 25 | T | 2.4 (NA) | NA |
|  | Anti-PD-1 +- anti-CTLA4 | 13 | T | 20.3 (NA) | NA |
| Schmidberge, 2018 [26] | ipilimumab after RT (REF) | 21 | T | 6 (NA) | 1 |
|  | ipilimumab before RT | 20 | T | 2 (NA) | 2.1 |
| Le Rhun, 2020 [19] | SRS without or with non-IO | 20 | T | 6 (4-22) | NA |
|  | SRS+IO | 32 | T | 5 (3-13) | NA |
|  | IO alone | 10 | T | 3 (1-4) | NA |
| Skrepnik, 2017 [27] | SRS+ipilimumab | 25 | T | 16.7 (NA) | NA |
| Minniti, 2019 [22] | SRS+IO concurrent nonconcurrent (REF) | 26 (total) | D | 3.4 (NA) | 1 |
|  | SRS+IO concurrent | 26 (total) | D | 19 (NA) | 0.2 (0.1-0.4) |

**Abbreviations:** Anti-CTLA-4 = Anti-cytotoxic T-lymphocyte associated protein 4; Anti-PD-1 = Anti-programmed cell death protein 1; BRAFi = BRAF inhibitor; IO = immunotherapy; MEKi = MEK inhibitor; NA = not reported; REF = reference; RT = radiation therapy; SRS = stereotactic radiosurgery; ^a^T, from start of treatment; D, from date of diagnosis

**Table S8. Clinical Trials Reported Median Overall Survival (OS) in Months (N=11) and Hazard Ratio (HR) for OS (N = 7)**

| **First author, Year** | **Treatment group** | **Number of patients** | **Median OS (Range, 95% CI or IQR)** | **HR (95% CI)** |
| --- | --- | --- | --- | --- |
| Davies, 2017 [44] | Dabrafenib + trametinib--BRAF V600E, symptomatic MBM, no prior local brain therapy | 76 | 10.8 (8.7-19.6)^b^ | NA |
|  | Dabrafenib + trametinib-BRAF V600E, asymptomatic MBM, prior local brain therapy | 16 | 24.3 (7.9-NR)^b^ |  |
|  | Dabrafenib + trametinib-BRAF V600D/K/R, asymptomatic MBM, with or without prior local brain therapy | 16 | 10.1 (4.6-17.6)^b^ |  |
|  | Dabrafenib + trametinib-BRAF V600D/E/K/R, symptomatic MBM, with or without prior local brain therapy | 17 | 11.5 (6.8-22.4)^b^ |  |
| Goldberg, 2016 [45] | pembrolizumab | 18 | NA | NA |
| Gupta, 2016 [46] | WBRT + placebo | 8 | 2.5 (0.2-7.2)^b^ | 1.0 |
|  | WBRT + vandetanib | 10 | 4.6 (1.6-6.3)^b^ | 0.85 (0.37-1.96) |
| Hauswald, 2019 [47] | WBRT | 3 | 4 (NA) | NA |
|  | WBRT with simultaneous integrated boost | 4 | 5 (NA) |  |
| Hoffmann-La Roche, 2016 [48] | Vemurafenib (previously untreated MBM): | 146 | 8.87 (0.59-34.53)^a^ | NA |
|  | Vemurafenib (previously treated MBM): |  | 9.63 (0.66-34.30)^a^ |  |
| Hong, 2019 [49] | observation after surgery and/or SRS | 107 | 13 (10-19)^b^ | 1.0 |
|  | WBRT after surgery and/or SRS | 101 | 16.5 (13-24)^b^ | 0.95 (0.69-1.31) |
| Kluger, 2019 [50] | pembrolizumab | 23 | 17 (10-NR)^b^ | NA |
| Long, 2018 [51] | nivolumab alone | 25 | 18.5 (6.9-NR)^b^ | 1.0 |
|  | nivolumab alone (those who failed local therapy) | 16 | 5.1 (1.8-NR)^b^ | 1.0 |
|  | nivolumab + ipilimumab | 35 | NR (8.5-NR)^b^ | 1.01 (0.43-2.4)^a^  0.42 (0.16-1.12)^b^ |
| McArthur, 2017 [52] | BRAFi (previously untreated MBM) | 90 | 8.9 (4.9-17.0)^c^ | 1.0 |
|  | BRAFi (previously treated MBM) | 56 | 9.6 (4.5-18.4)^c^ | 0.95 (0.66-1.36) |
| McQuade, 2016 [53] | temozolomide+TPI287 | 10 | 6.1 (3.7-NR)^b^ | NA |
| Tawbi, 2018 [54] | Nivolumab + Ipilimumab | 94 | NR (81.5% OS at 12 months) | NA |
| Squibb, 2014 [55] | No reference group listed |  | NA | 1.0 |
|  | Ipilimumab, 10 mg/kg IV, in Corticosteroid-free Patients |  | NA | 0.31(0.18-0.44) |
|  | Ipilimumab, 10 mg/kg IV, in Corticosteroid-dependent Patients |  | NA | 0.19 (0.02-0.36) |

**Abbreviations:** BRAFi = BRAF inhibitor; MBM = melanoma brain metastases; NA = not reported; NR = not reached; SRS = stereotactic radiosurgery; WBRT = whole brain radiation therapy

^a^ Range provided in the study

^b^ 95% CI provided in the study

^c^ IQR provided in the study

**Table S9. Hazard Ratios (HR) for Prognostic Factors of Overall Survival (OS) among Patients with MBM**

| **Prognostic Factors** | **First Author, Year** | **Comparison Groups** | **HR (95% CI)** |
| --- | --- | --- | --- |
| LDH | Gummadi, 2015 [56] | High vs Normal^a^ | 2.40 (1.57-3.67) |
|  | Partl, 2016 [57] | Elevated vs Normal^b^ | 2.30 (1.60-3.30) |
|  | Tio, 2018 [42] | >ULN vs ≤ULN | 1.56 (0.94-2.57) |
|  | Amaral, 2020 [39] | Elevated vs Normal | 1.05 (0.69-1.59) |
|  | Geukes Foppen, 2018 [18] | >ULN vs ≤ULN | 1.30 (0.80-2.10) |
| Sex | Gummadi, 2015 [56] | Male vs Female | 1.36 (0.99-1.87) |
|  | Chowdhury, 2015 [58] |  | 1.76 (1.04-2.97) |
|  | Bian, 2016 [59] |  | 1.36 (1.08-1.71) |
|  | Rodenburg, 2018 [60] |  | 1.64 (1.05-2.56) |
|  | Ramos, 2020 [61] |  | 0.42 (0.15-1.19) |
|  | Amaral, 2020^c^ [39] | Male vs Female | 0.74 (0.67-1.43) |
| BRAF | Gallaher, 2016 [62] | Mutated vs Wild-type | 0.2 (0.1–0.7) |
|  | Sperduto, 2017 [63] |  | 0.81(0.68-0.96) |
|  | Xu, 2017 [64] |  | 0.58 (0.39-0.84) |
|  | Schaule, 2020 [65] |  | 0.58 (0.32-1.05) |
|  | Frinton, 2017 [66] |  | 0.54 (0.25-1.19) |
|  | Mastorakos, 2019 [67] |  | 0.58 (0.39-0.84) |
|  | Zhang, 2020 [68] |  | 0.77 (0.20-3.01) |
|  | Tio, 2018 [37] |  | 0.57 (0.31-1.05) |
|  | Amaral, 2020 [39] |  | 1.13 (0.76-1.67) |
|  | Maxwell, 2017 [69] | V600 mutation w/o BRAFi vs Wild-type | 0.66 (0.36-1.20) |
|  |  | V600 mutation w/ BRAFi vs Wild-type | 0.30 (0.11-0.79) |
| Intracranial Metastases | Badakhshi, 2018 [70] | MBM: >1 vs 1 | 3.55 (1.71-7.35) |
|  | Zubatkina, 2018 [71] |  | 1.35 (0.63-2.92) |
|  | Schaule, 2020 [65] |  | 0.67 (0.20-2.35) |
|  | Chowdhury, 2015 [58] | MBM: 2-4/5 vs 1 | 2.61 (1.37-4.96) |
|  | Bian, 2016 [59] |  | 1.25 (0.99-1.58) |
|  | Ferrel, 2016 [72] |  | 1.04 (0.61-1.79) |
|  | Rodenburg, 2018 [60] |  | 1.53 (0.93-2.51) |
|  | Sloot, 2018 [34] |  | 1.52 (0.92-2.52) |
|  | Chowdhury, 2015 [58] | MBM:≥ 4/5 vs 1 | 3.24 (1.56-6.72) |
|  | Bian, 2016 [59] |  | 2.18 (1.56-3.03) |
|  | Ferrel, 2016 [72] |  | 2.30 (2.09-2.53) |
|  | Sloot, 2018 [34] |  | 1.95 (1.04-3.66) |
|  | Rodenburg, 2018 [60] |  | 1.41 (0.79-2.53) |
|  | Kavouridis, 2019 [73] |  | 2.33 (1.32-4.11) |
|  | Ferrel, 2016 [72] | MBM: unknown vs. 1 | 2.98 (1.16-7.68) |
|  | Hirshman, 2018 [74] | MBM: larger vs smaller number (not specified) | 1.00; *P* = 0.96 |
|  | Kano, 2018 [75] | Brain tumors: larger vs smaller number | 1.07 (1.04-1.09) |
|  | Tio, 2018 [37] | >3 vs 1-3 | 1.28 (0.69-2.36) |
|  | Amaral, 2020 [39] | >3 vs 1-3 | 1.67 (1.14-2.44) |
|  | Geukes Foppen, 2018 [18] | >5 vs ≤5 | 1.6 (1.0-2.5) |
|  | Bates, 2015 [76] | > 2 vs ≤2 | 1.66 (1.00-2.76) |
|  | Wilkins, 2015 [77] | MBM: 1 vs >3^d^ | 0.43 (0.27-0.70) |
|  |  | MBM: 2-3 vs >3^d^ | 0.88 (0.53-1.44) |
| Extracranial Metastases | Bates, 2015 [76] | Active vs. Controlled | 1.70 (0.68-4.27) |
|  | Chowdhury, 2015 [58] |  | 5.37 (1.65-17.48) |
|  | Kano, 2018 [75] |  | 2.20 (1.73-2.81) |
|  | Rodenburg, 2018 [60] |  | 1.93 (1.19-3.14) |
|  | Schaule, 2020 [65] |  | 1.35 (0.37-4.98) |
|  | Zubatkina, 2018 [71] |  | 2.75 (1.49-5.09) |
|  | Zhang, 2020 [68] |  | 1.22 (0.37-3.90) |
|  | Tio, 2018 [42] |  | 1.14 (0.85-1.53) |
|  | Wilkins, 2015 [77] | 3 vs ≥ 4 | 0.64 (0.39-1.06) |
|  |  | 2 vs ≥ 4 | 0.34 (0.20-0.61) |
|  |  | 1 vs ≥ 4 | 0.29 (0.17-0.52) |
|  |  | 0 vs ≥ 4 | 0.22 (0.10-0.50) |
|  | Rades, 2016 [78] | 1 vs 0 | 1.27 (1.02-1.56) |
| KPS | Partl, 2016 [57] | ≤70 vs >70 | 4.4 (3.0-6.4) |
|  | Rades, 2016 [78] |  | 2.11 (1.28-3.47) |
|  | Frinton, 2017 [66] |  | 2.18 (0.83-5.70) |
|  | Sehmisch, 2017 [79] |  | 2.09 (0.98-4.46) |
|  | Wilkins, 2015 [77] | ≤70 vs >90-100 | 2.86 (1.75-4.76) |
|  | Sloot, 2018 [34] |  | 2.41 (1.19-4.86) |
|  | Chowdhury, 2015 [58] | > 80 vs ≤80 | 8.09 (3.79-17.28) |
|  | Rodenburg, 2018 [60] |  | 2.39 (1.49-3.82) |
|  | Bates, 2015 [76] | ≥90 vs <90 | 2.86 (1.67-5.0) |
|  | Badakhshi, 2018 [70] |  | 3.99 (1.78-8.96) |
|  | Kano, 2018 [75] | lower vs higher | 0.99 (0.98-0.99) |
|  | Zubatkina, 2018 [71] |  | 1.06 (1.03-1.11) |
|  | Kavouridis, 2019 [73] |  | 1.04 (1.02-1.05) |
|  | Ferrel, 2016 [72] | 70-80 vs 90-100 | 1.67 (1.20-2.34) |
|  |  | <=60 vs 90-100 | 5.56 (5.17-5.97) |
|  |  | unknown vs 90-100 | 2.43 (0.85-6.90) |
|  | Zhang, 2020 [68] | 90 vs 100 | 0.65 (0.10-4.16) |
|  |  | 80 vs 100 | 2.42 (0.31-18.79) |
|  |  | 70 vs 100 | 3.36 (0.29-38.23) |
|  |  | Unknown vs 100 | 1.77 (0.28-11.30) |
|  | Sloot, 2018 [34] | >70-90 vs >90-100 | 1.08 (0.65-1.78) |
|  | Hirshman, 2018 [74] | lower vs higher | 1.14, p=0.36 |
| Brain Metastases Volume | Kano, 2018 [75] | Larger vs smaller (not specified) | 1.02 (1.01-1.03) |
|  | Zubatkina, 2018 [71] |  | 1.01 (0.96-1.05) |
|  | Bian, 2016 [59] | 5-10 cm^3^ vs <5 cm^3^ | 1.33 (1.01-1.77) |
|  |  | >10 cm^3^ vs <5 cm^3^ | 1.66 (1.23-2.25) |
|  | Badakhshi, 2018 [70] | <median vs ≥ median (2.47 cm^3^) | 1.0 (0.94-1.06) |
| Leptomeningeal Disease | Tio, 2018 [37] | Present vs Absent | 1.93 (0.22-16.90) |
|  | Wilkins, 2015 [77] |  | 2.45 (0.95-6.33) |
| Age | Gummadi, 2015 [56] | Continuous | 1.00 (0.99-1.01) |
|  | Marzese, 2015 [80] |  | 1.02 (0.99-1.06) |
|  | Wilkins, 2015 [77] |  | 1.01 (0.99-1.02) |
|  | Farris, 2017 [81] |  | 1.02 (1.01-1.03) |
|  | Ramos, 2020 [61] |  | 1 (0.97-1.04) |
|  | Mastorakos, 2019 [67] |  | 1.02 (1.01-1.04) |
|  | Liu, 2019 [59] |  | 1.01 (1.00-1.09) |
|  | Tio, 2018 [42] | ≥ 65 vs <65 years | 1.11 (0.64-1.95) |
|  | Ferrel, 2016 [72] |  | 1.03 (0.60-1.77) |
|  | Rades, 2016 [78] | >61 vs ≤ 61 years | 1.46 (0.90-2.38) |
|  | Schaule, 2020 [65] | >=70 vs <70 years | 1.32 (0.67-2.63) |
|  | Sehmisch, 2017 [79] | >=56 vs ≤ 55years | 3.40 (1.55-7.88) |
|  | Bian, 2016 [59] | >=50 vs <50 years | 1.29 (1.01-1.64) |

**Abbreviations:** BRAFi = BRAF inhibitor; KPS = Karnofsky Performance Scale; LDH = Lactate Dehydrogenase; MBM = melanoma brain metastases; NI = not included in meta-analysis; ULN = upper limit of normal

^a^Normal vs. High LDH = Definition or specific numerical cutoff of “high” not provided in manuscript

^b^Normal vs. Elevated LDH (according to individual lab reference ranges) = Definition of elevated value specified by the individual laboratory, and patients had results drawn from different laboratories

^c^The data did not look correct from the publication (point estimate was not central in the CI), thus we excluded it from the meta-analysis.

^d^Of note, the reference group in this study has higher number of MBM, compared to the other studies in this group which had lower numbers as the reference group

**Figure S1. Meta HR for SRS+MAPKi+IO vs SRS alone for OS starting point of treatment**

**
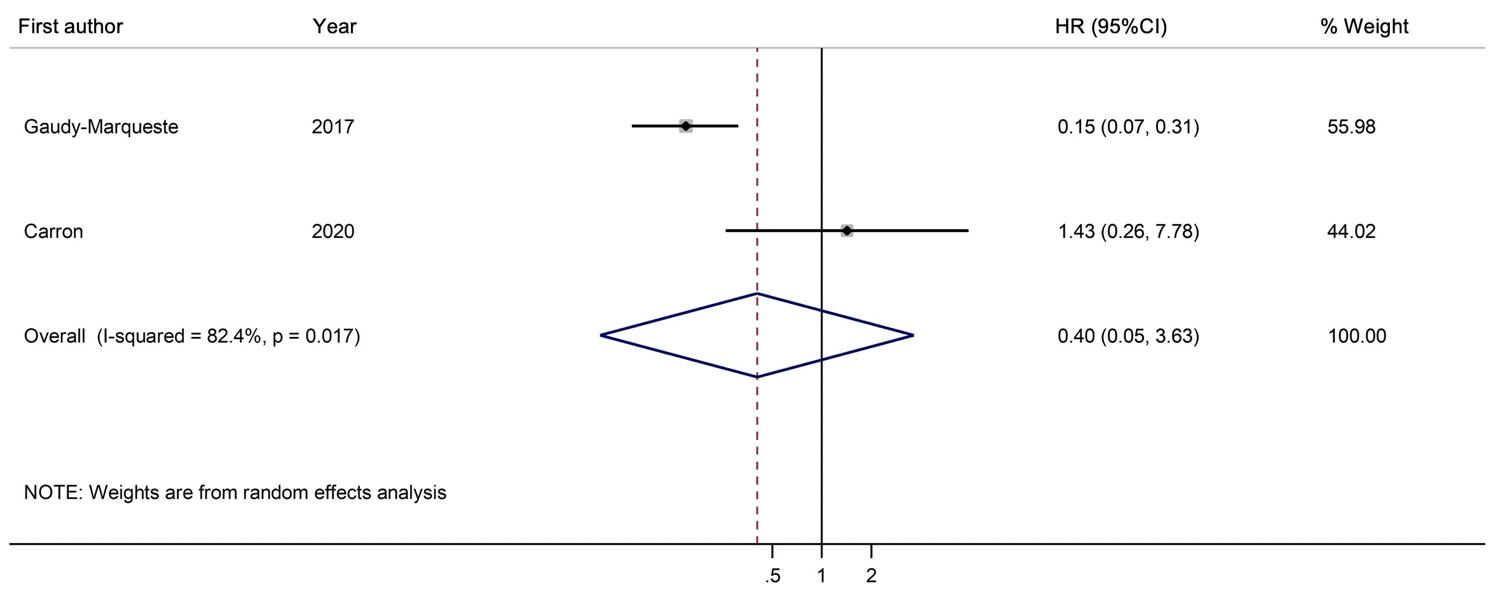
**

**Figure S2. Meta HR for SRS+IO vs SRS alone, separated by the OS starting point of treatment or diagnosis**

^
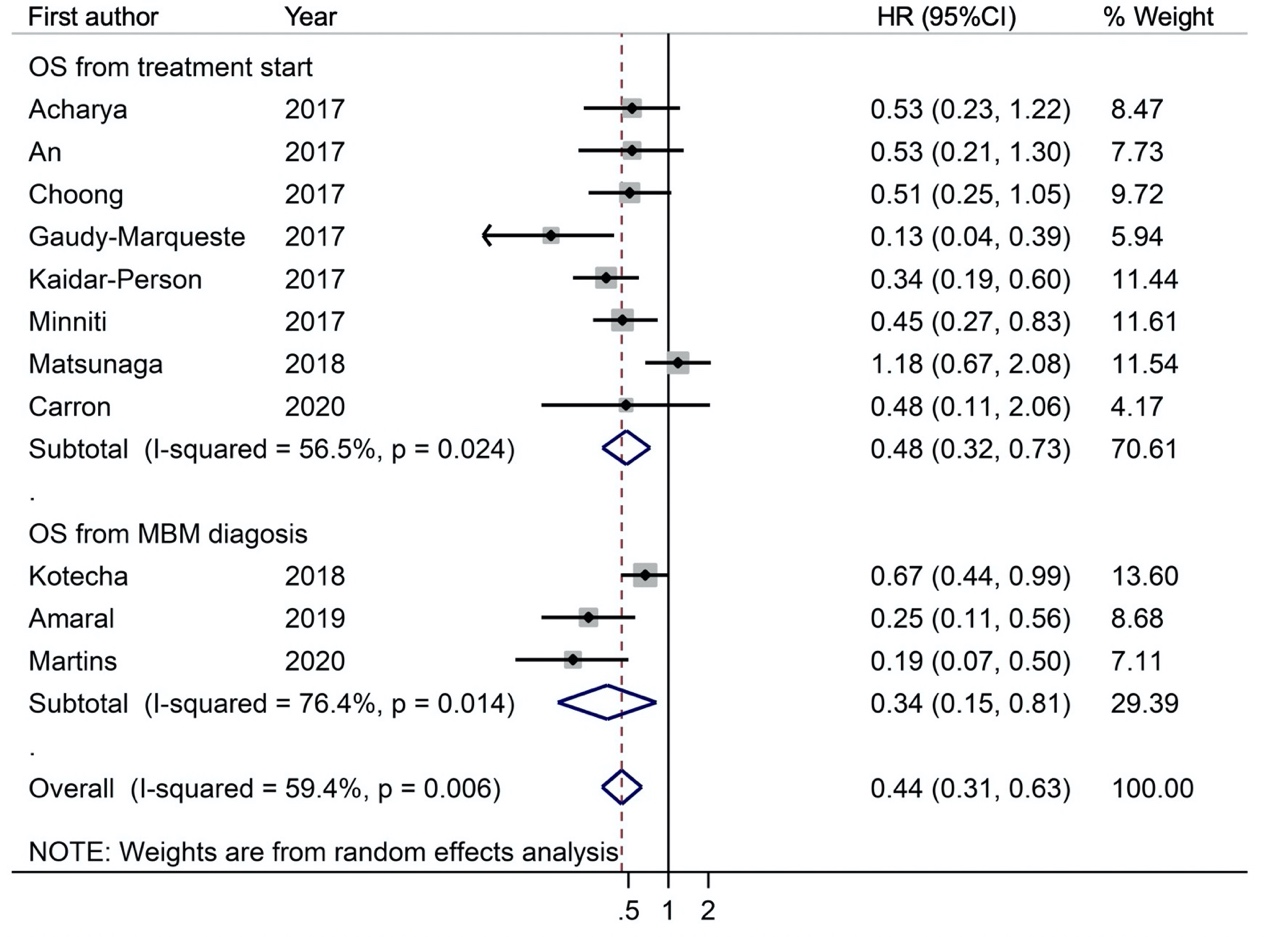
^

*Diao 2018 was not included due to two reported non-independent estimates.

**Figure S3. Meta HR for MAPKi vs. no MAPKi, separated by the OS starting point of treatment or diagnosis**

**
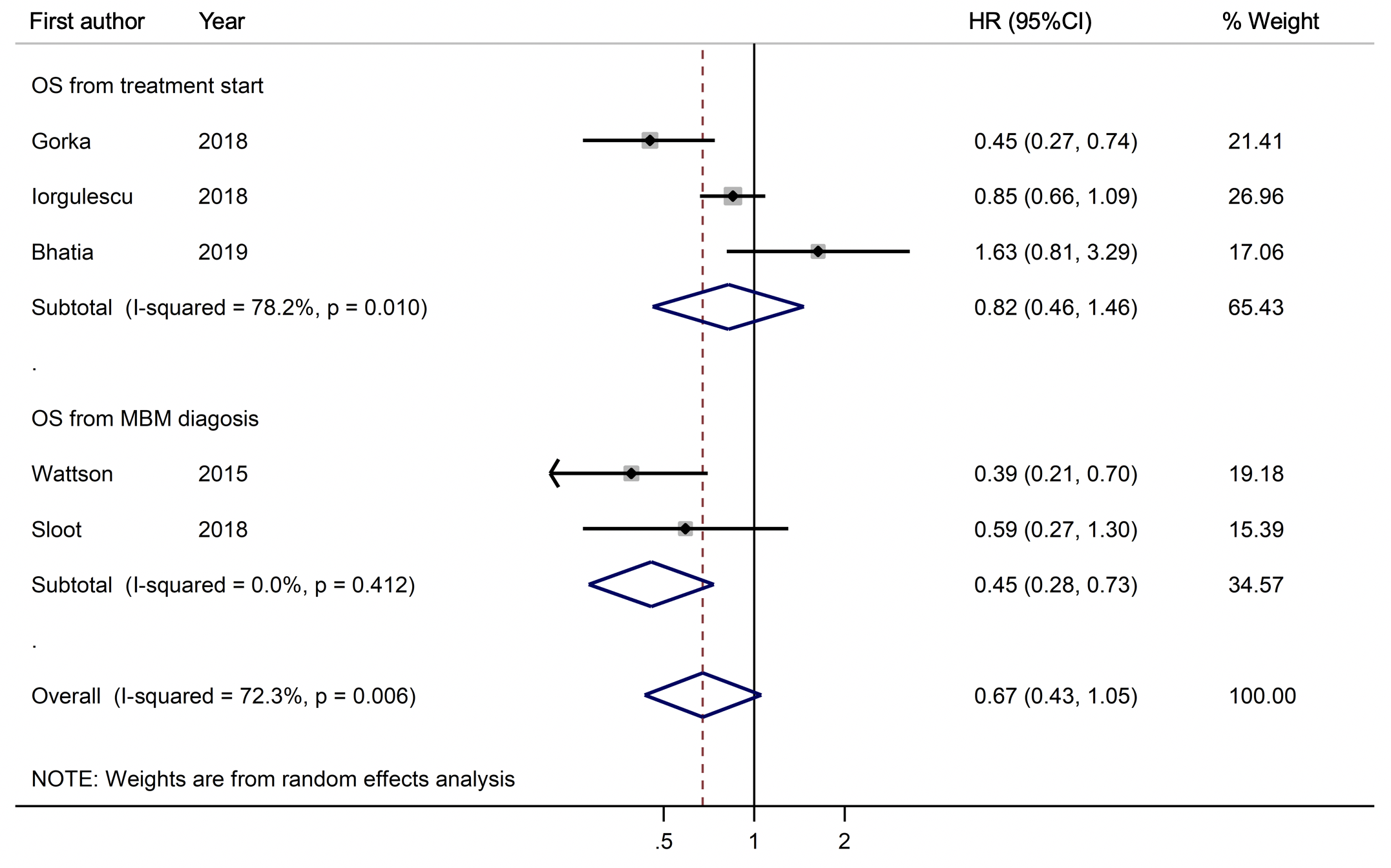
**

**Figure S4. Meta HR for SRS+MAPKi vs SRS alone, separated by the OS starting point of treatment or diagnosis**

**
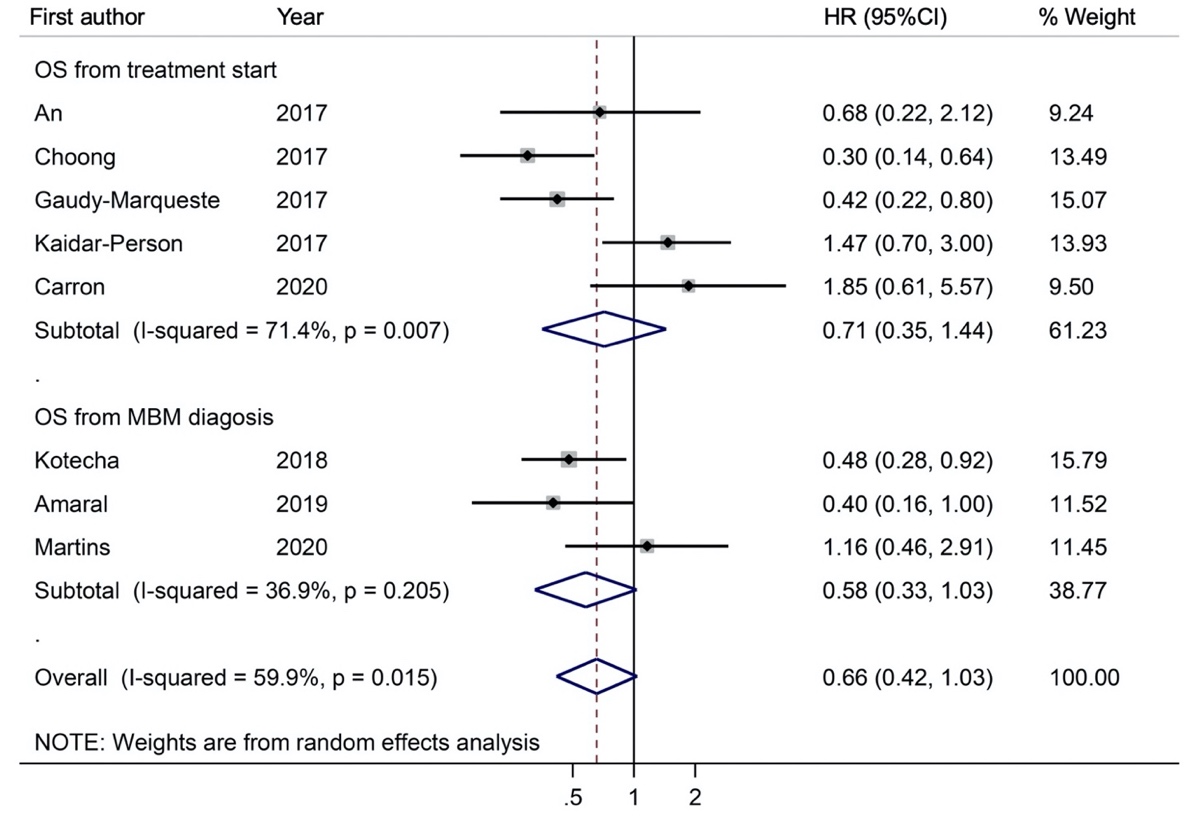
**

**Figure S5.** **Meta HR for SRS vs WBRT, separated by the OS starting point of treatment or diagnosis**

**
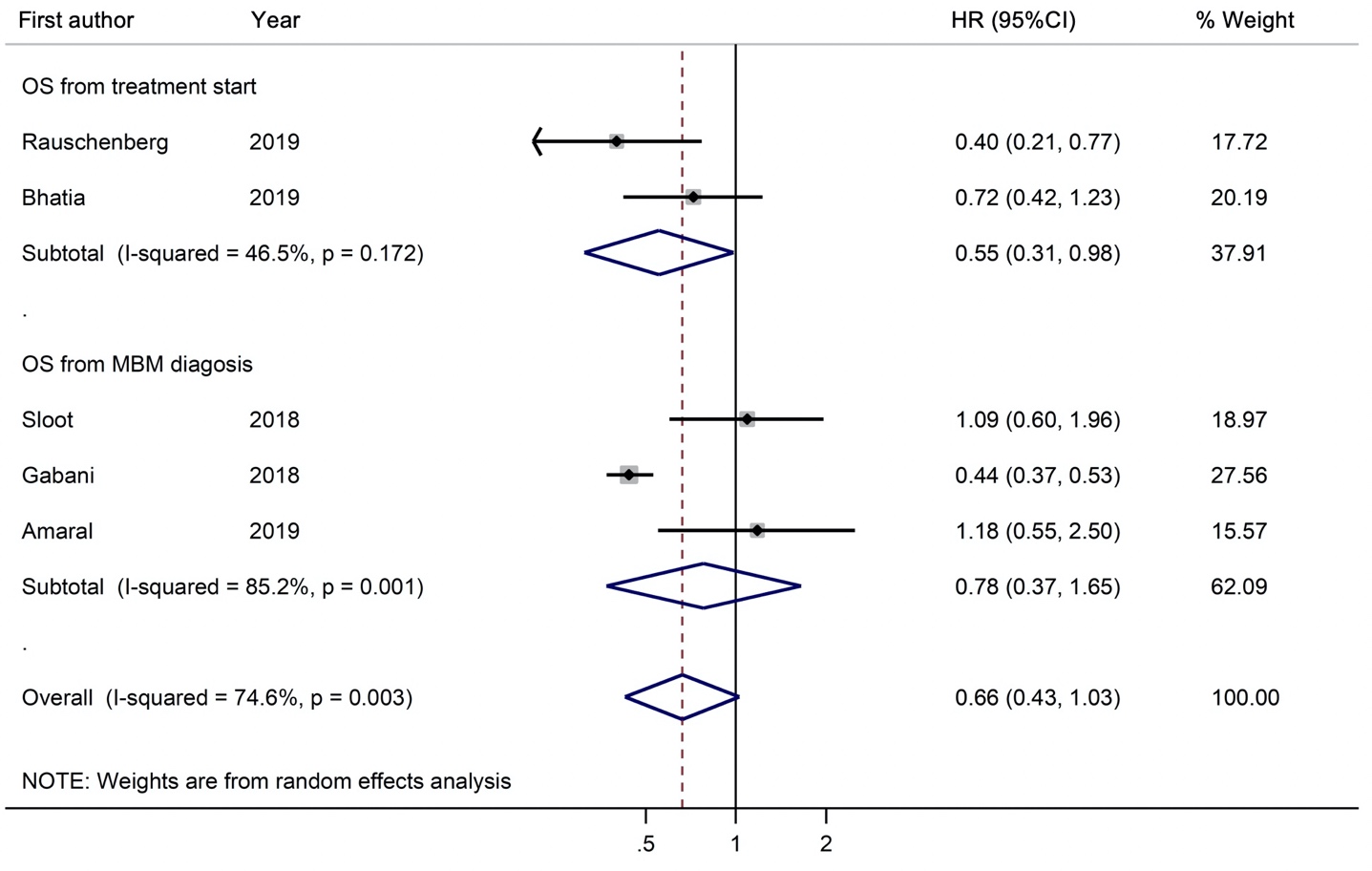
**

**Figure S6. Meta HR for IO vs. no IO for OS starting point of diagnosis**

**
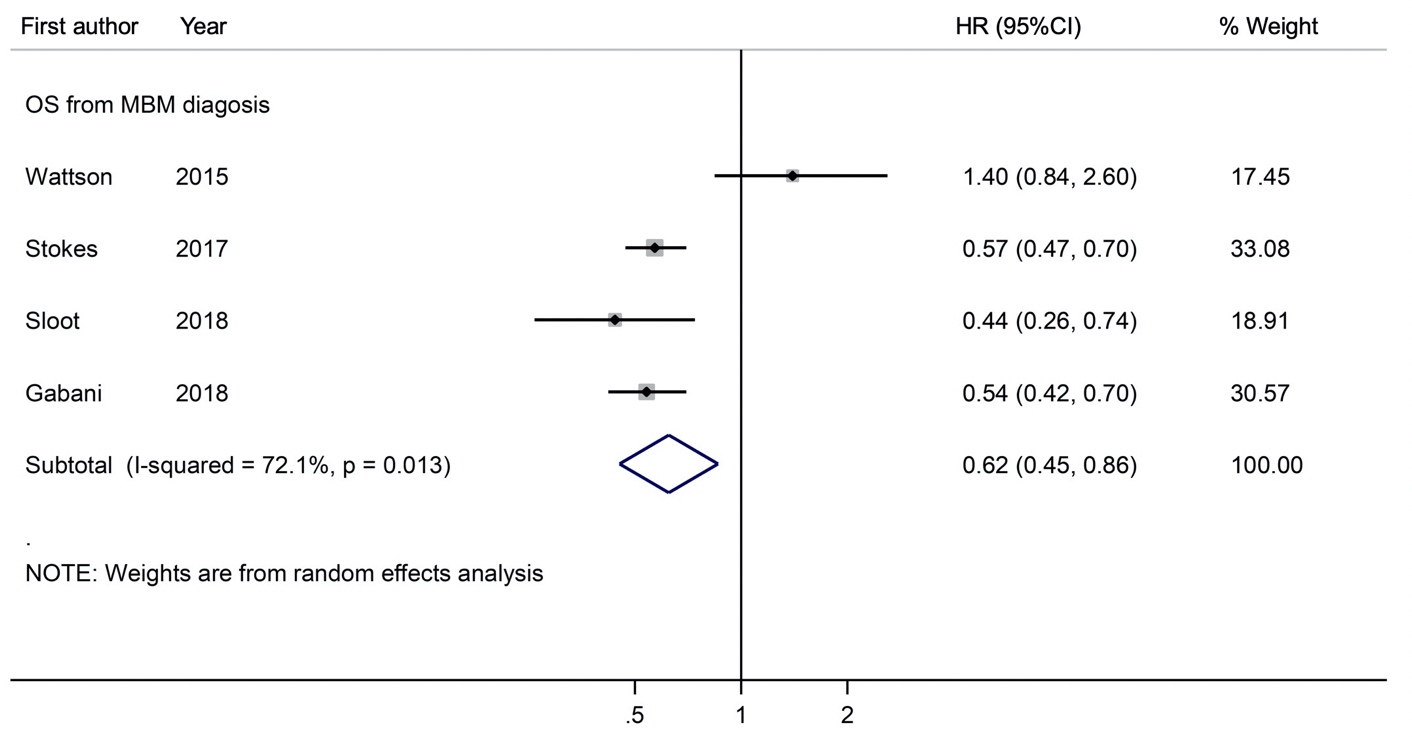
**

**Figure S7. Meta-analysis of LDH on OS**


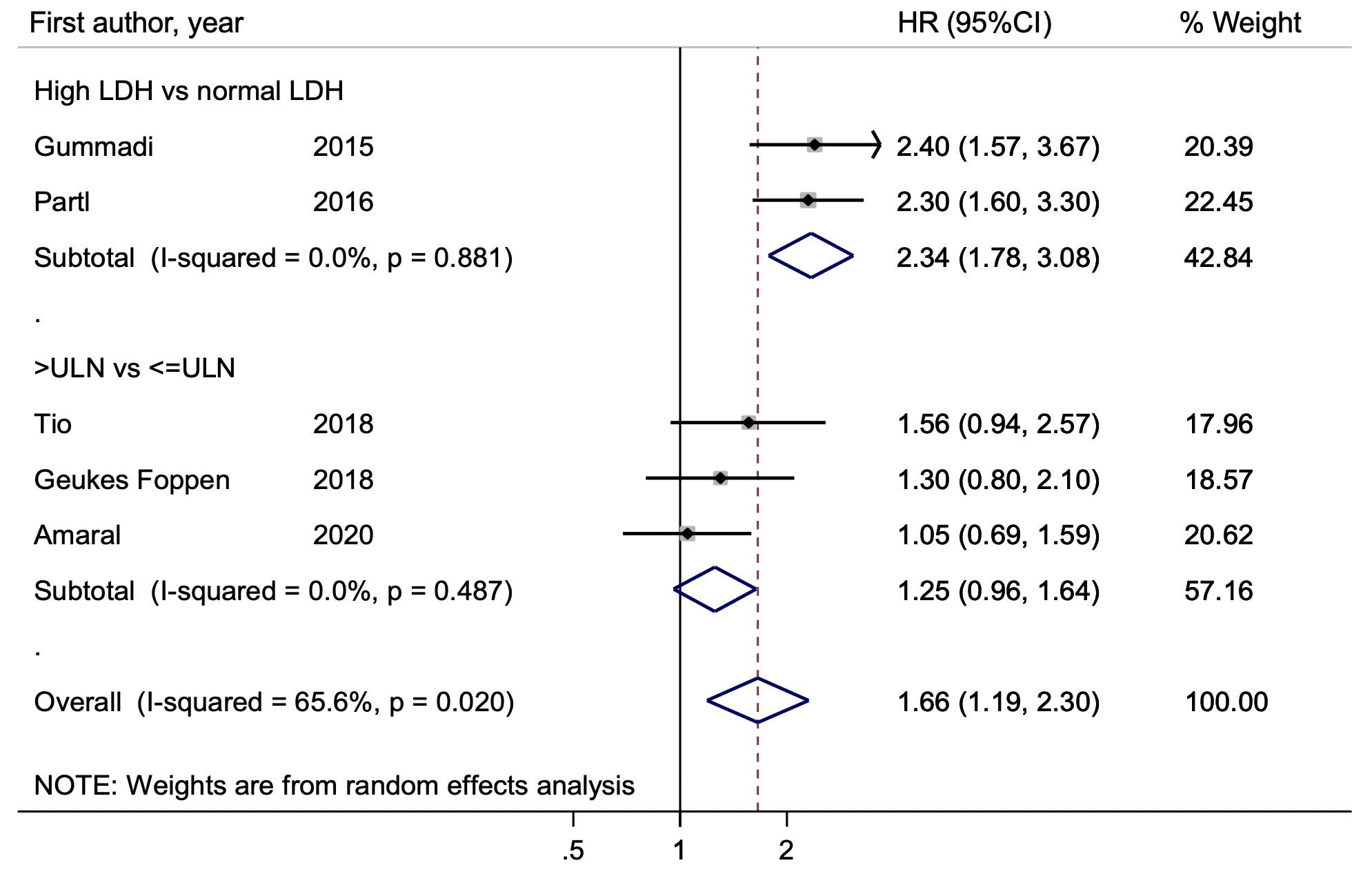


**Figure S8.** **Meta-analysis of gender on OS (Male vs. Female)**


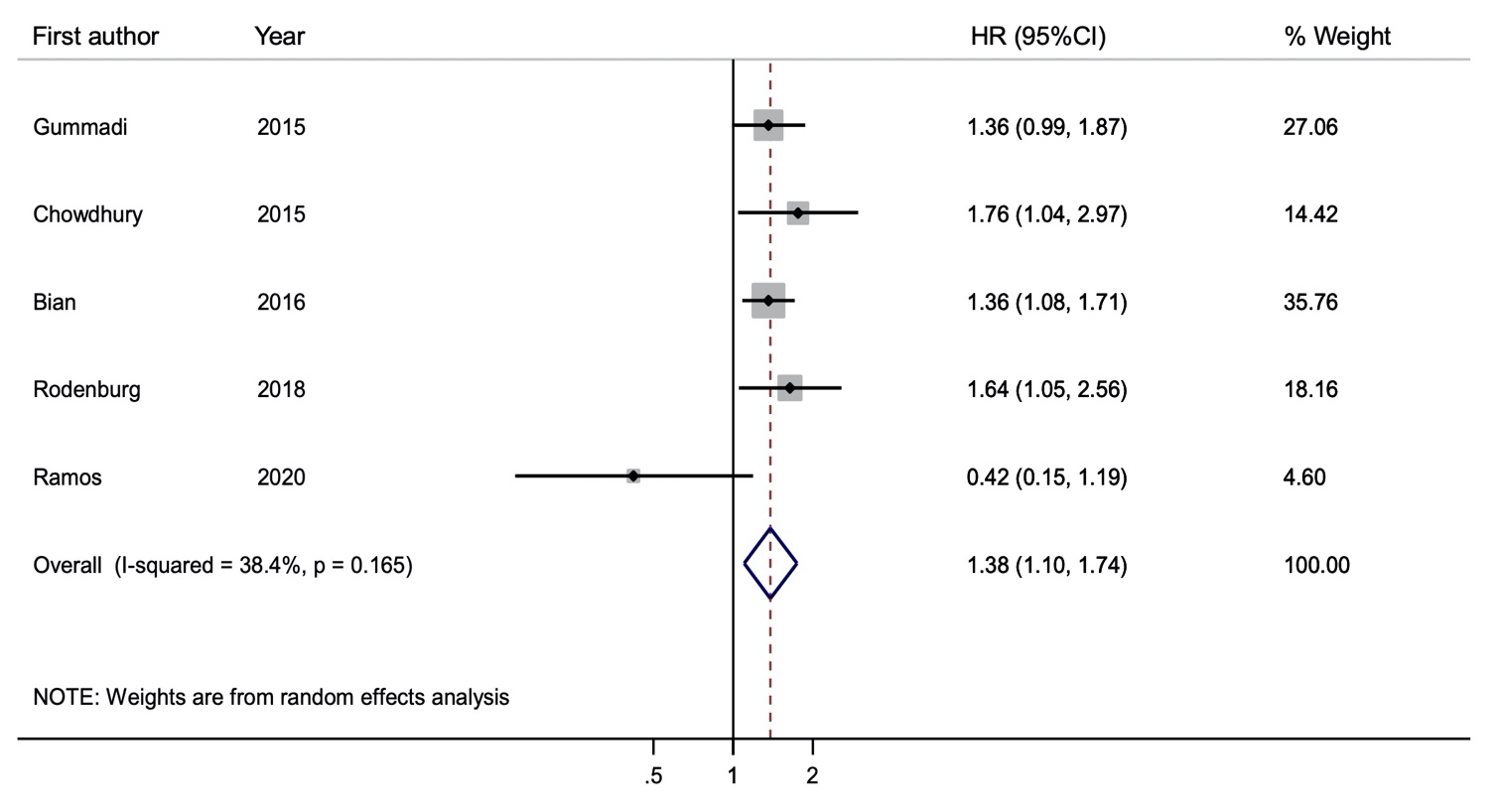


**Figure S9.** **Meta-analysis of BRAF on OS (Mutated vs. Wildtype)**


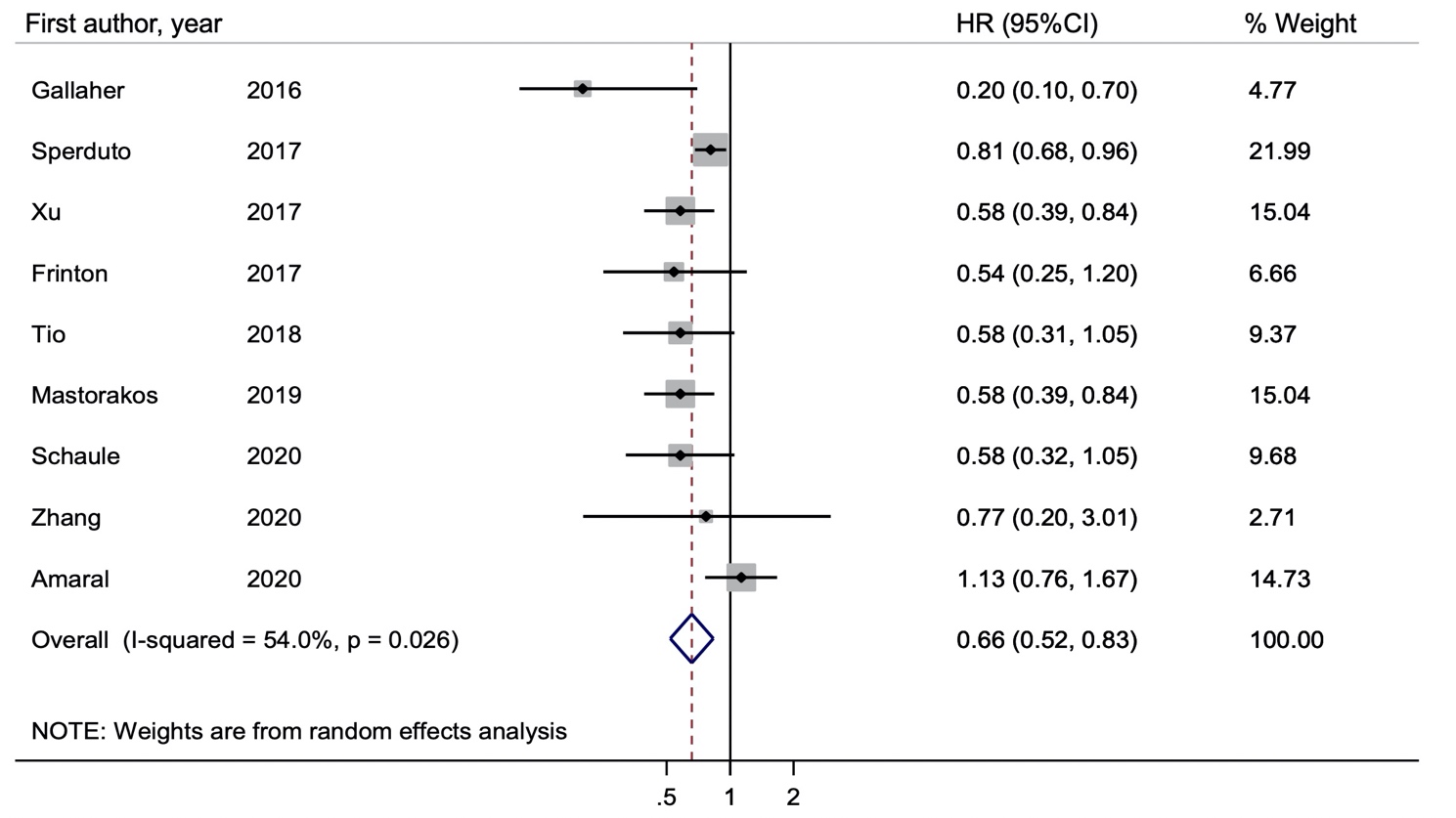


**Figure S10.** **Meta-analysis of number of intracranial metastases on OS**


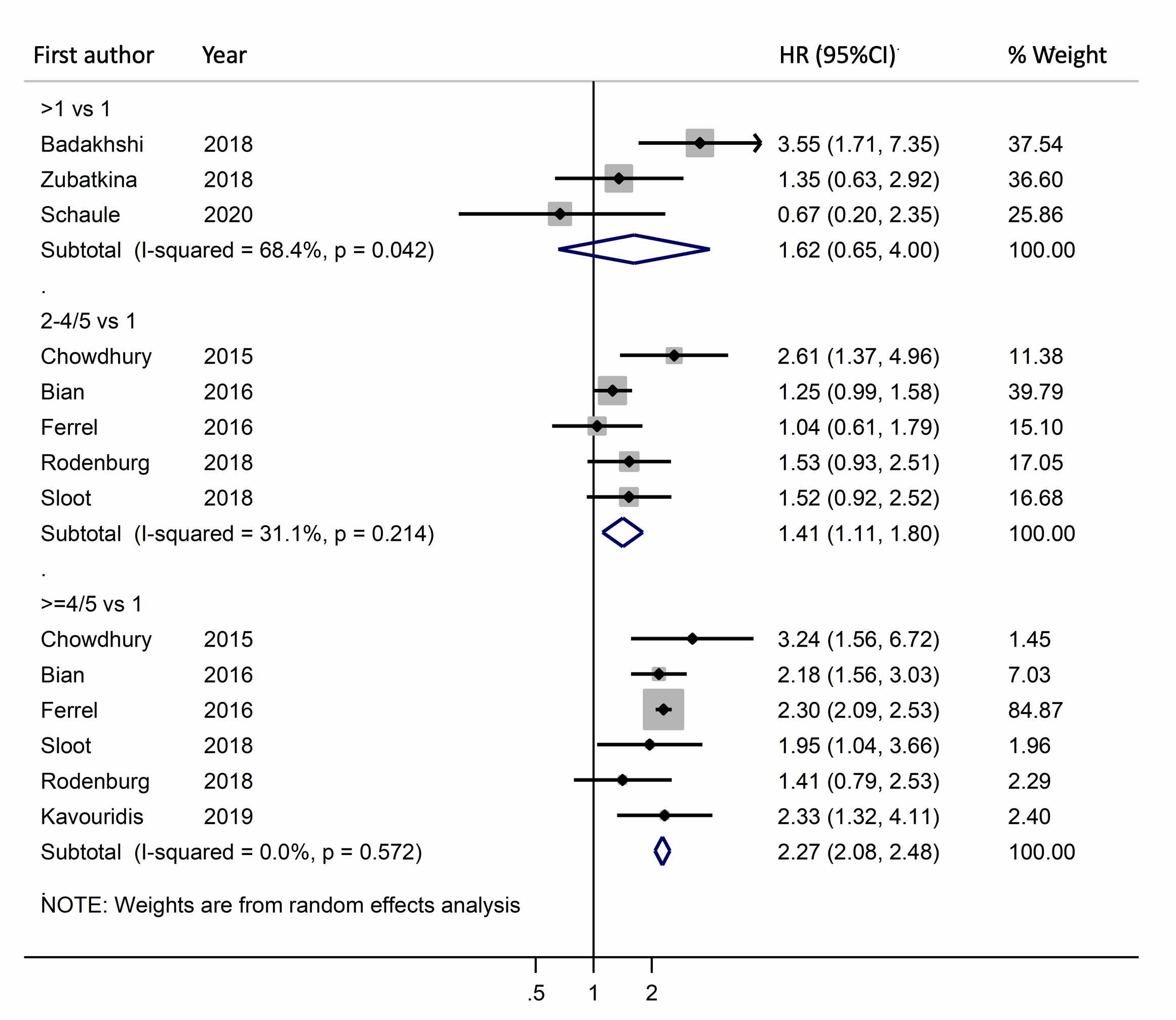


**Figure S11.** **Meta-analysis of Extracranial Metastases on OS (Active vs. Controlled)**


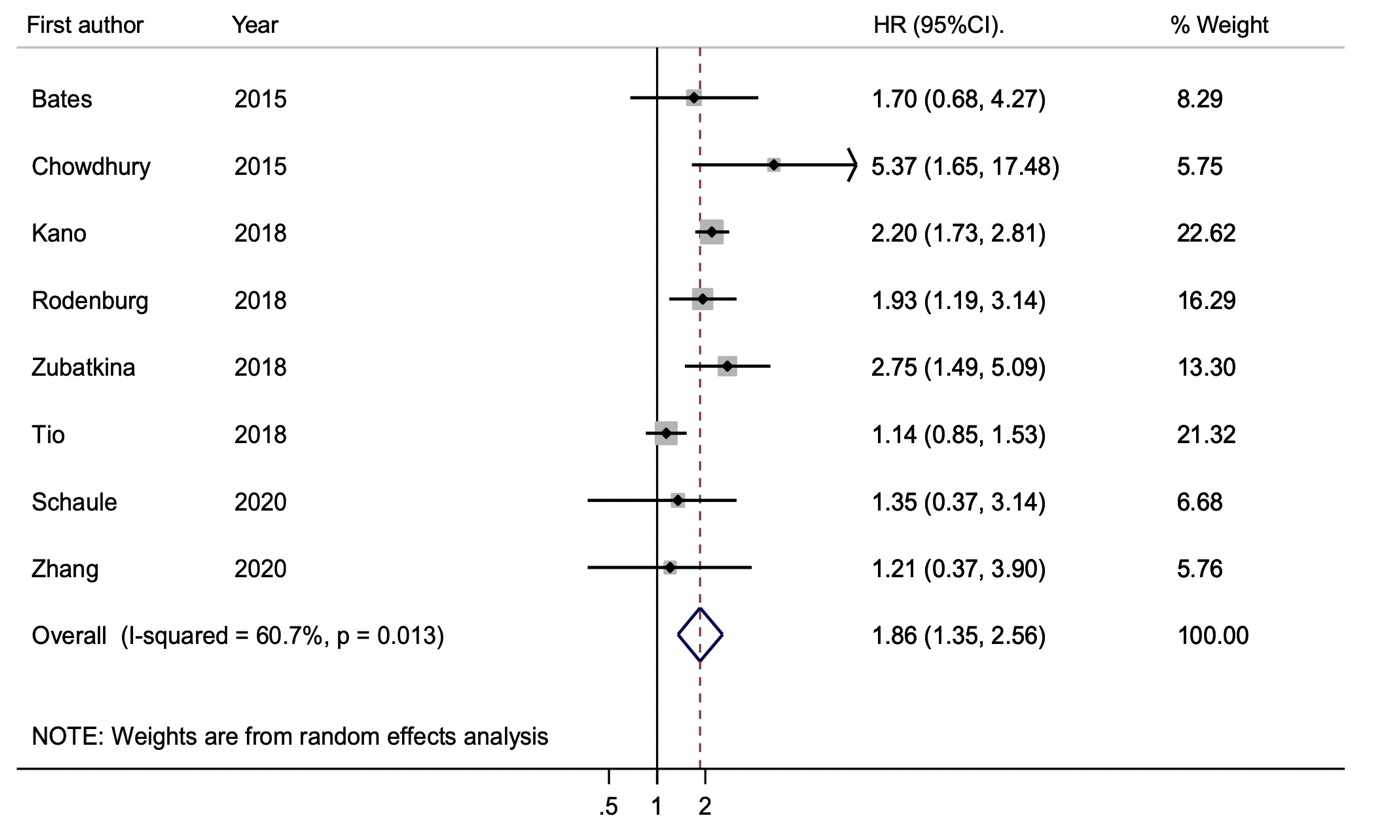


**Figure S12.** **Meta-analysis of KPS on OS**


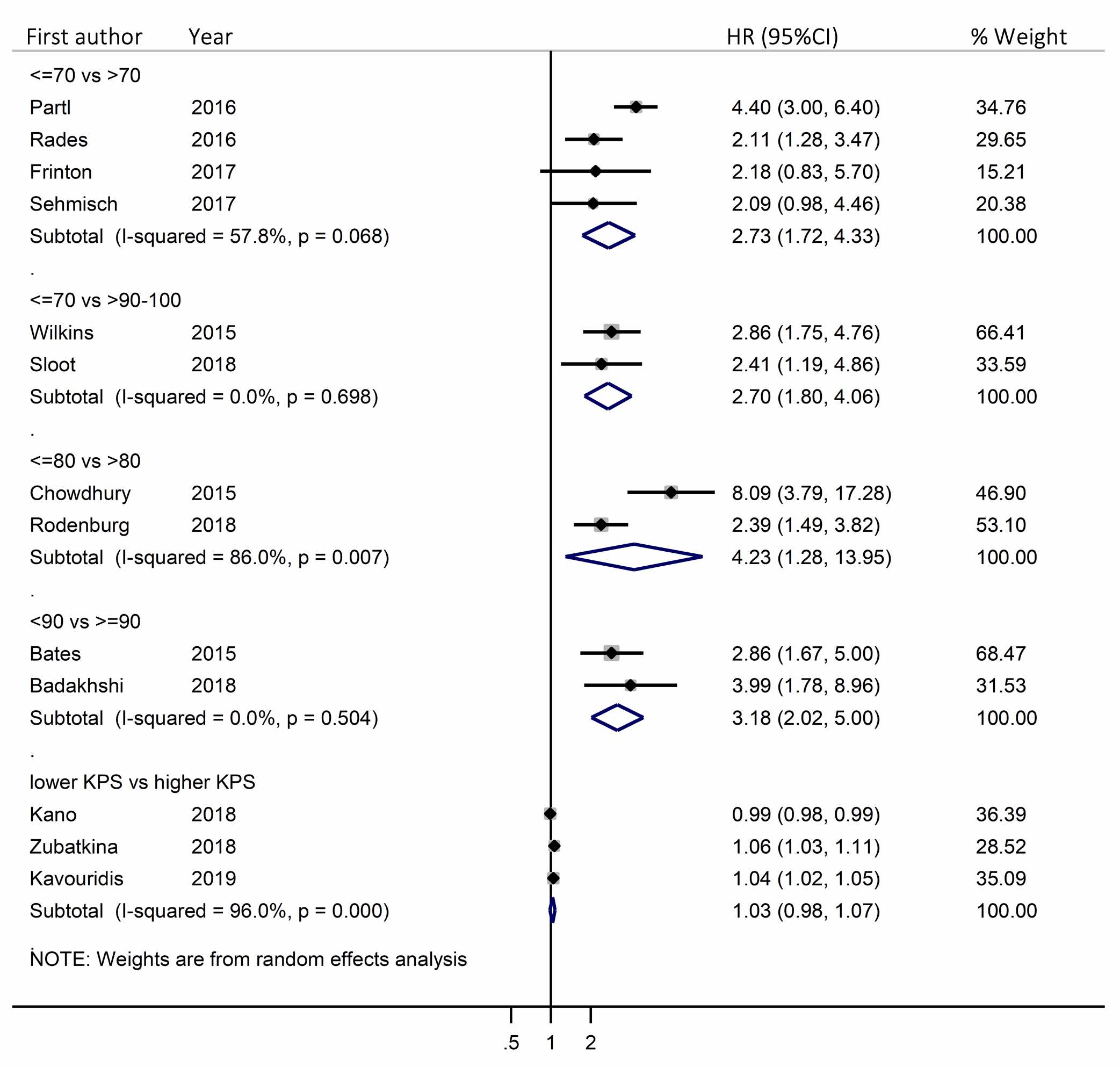


**Figure S13.** **Meta-analysis of brain metastases volume on OS**


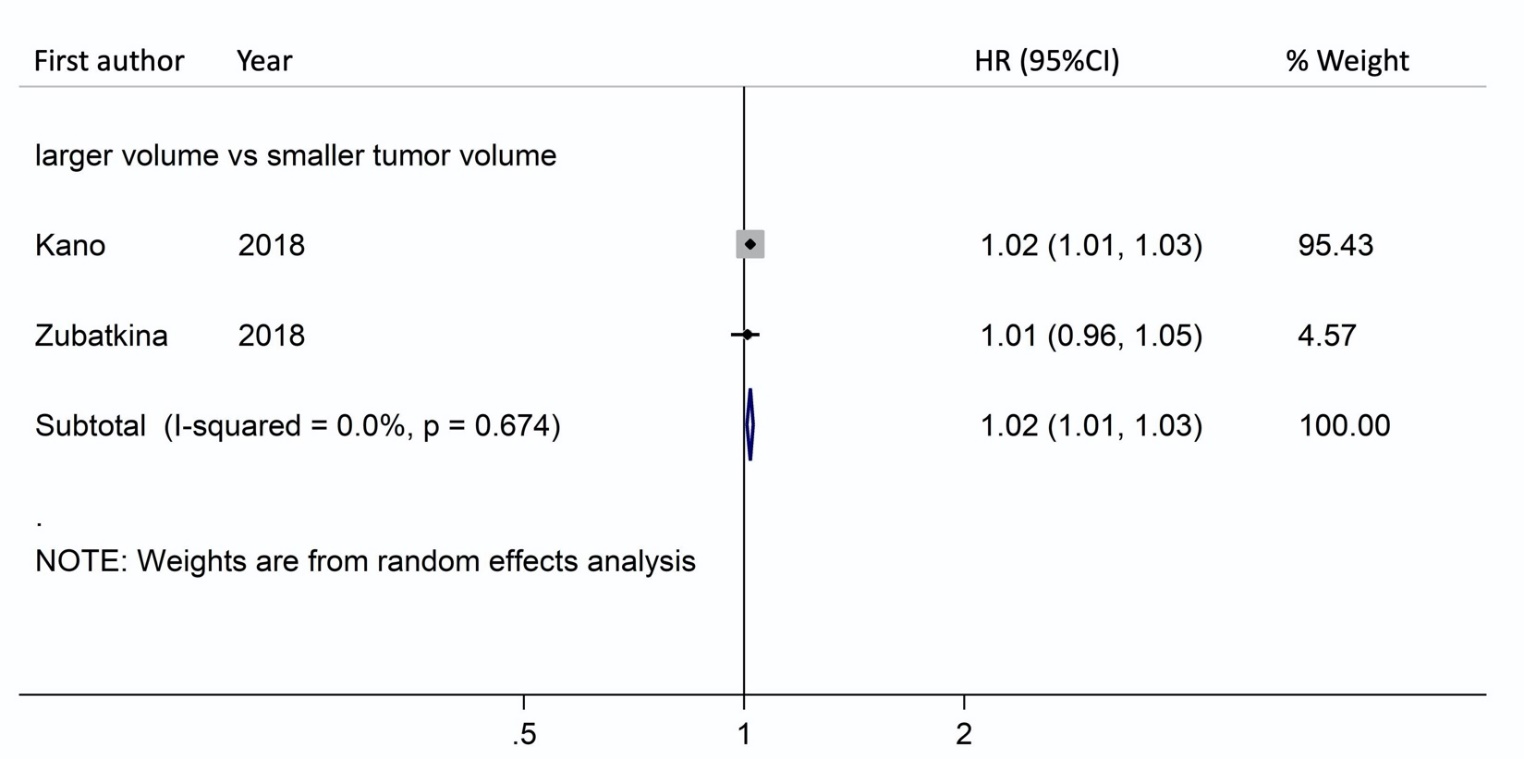


**Figure S14.** **Meta-analysis of Leptomeningeal Disease on OS (Present vs. Absent)**


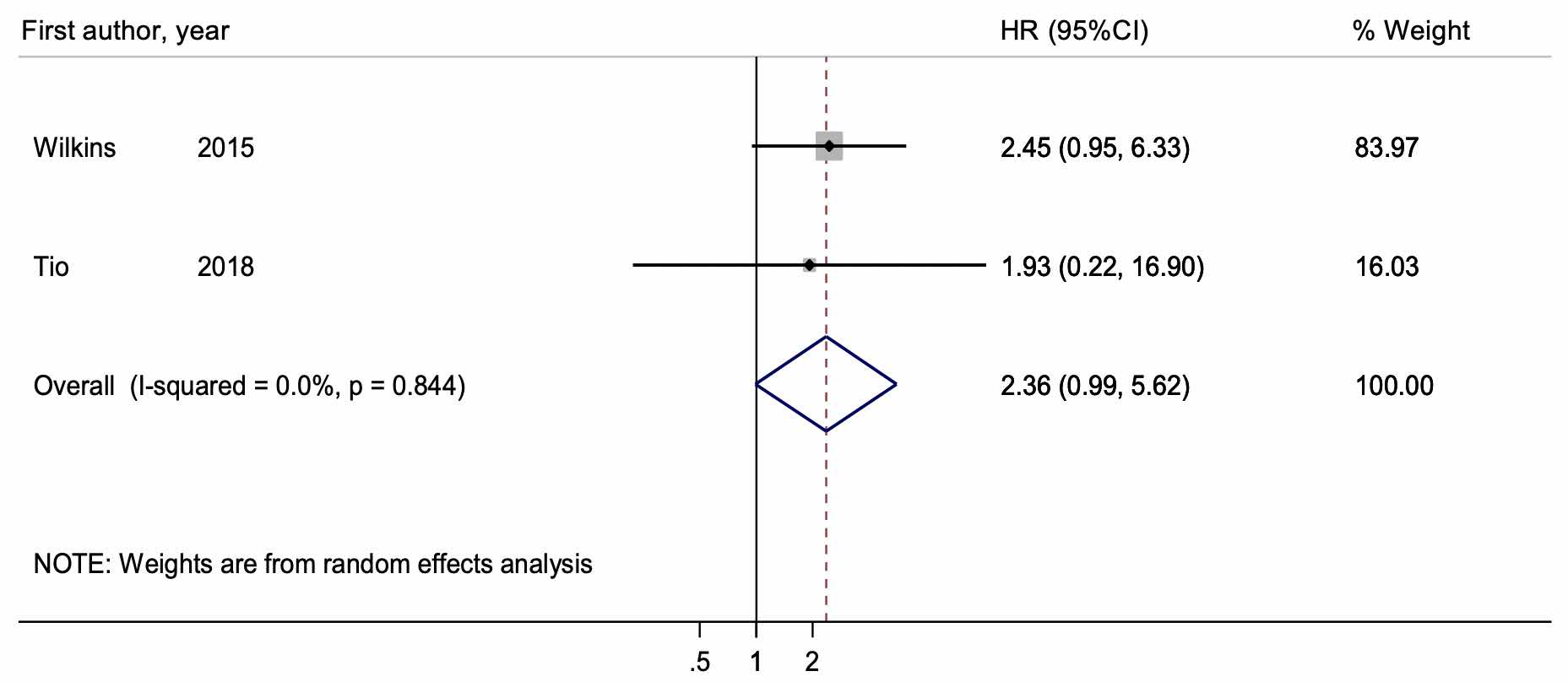


**Figure S15.** **Meta-analysis for the studies on association between age and OS**
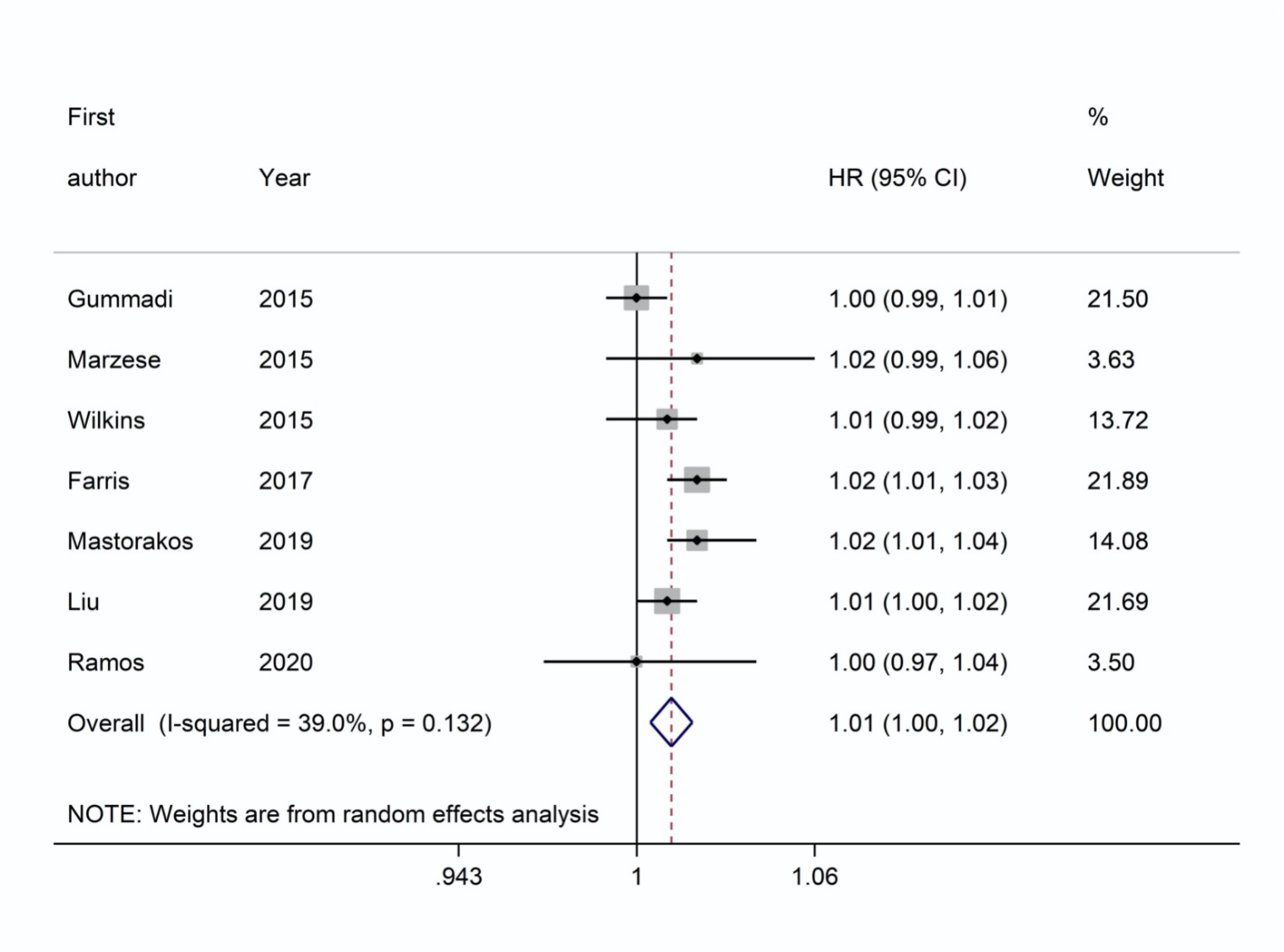


**References:**

1. Acharya, S., et al., *Distant intracranial failure in melanoma brain metastases treated with stereotactic radiosurgery in the era of immunotherapy and targeted agents.* Advances in Radiation Oncology, 2017. **2**(4): p. 572-580.

2. An, Y., et al., *Stereotactic radiosurgery of early melanoma brain metastases after initiation of anti-CTLA-4 treatment is associated with improved intracranial control.* Radiotherapy and Oncology, 2017. **125**(1): p. 80-88.

3. Carron, R., et al., *Stereotactic radiosurgery combined with anti-PD1 for the management of melanoma brain metastases: A retrospective study of safety and efficacy.* European Journal of Cancer, 2020. **135**: p. 52-61.

4. Choong, E.S., et al., *Survival of patients with melanoma brain metastasis treated with stereotactic radiosurgery and active systemic drug therapies.* European Journal of Cancer, 2017. **75**: p. 169-178.

5. Diao, K., et al., *Stereotactic radiosurgery and ipilimumab for patients with melanoma brain metastases: clinical outcomes and toxicity.* Journal of Neuro-Oncology, 2018. **139**(2): p. 421-429.

6. Gaudy-Marqueste, C., et al., *Survival of melanoma patients treated with targeted therapy and immunotherapy after systematic upfront control of brain metastases by radiosurgery.* European Journal of Cancer, 2017. **84**: p. 44-54.

7. Kaidar-Person, O., et al., *The incidence of radiation necrosis following stereotactic radiotherapy for melanoma brain metastases: The potential impact of immunotherapy.* Anti-Cancer Drugs, 2017. **28**(6): p. 669-675.

8. Matsunaga, S., et al., *Gamma Knife Radiosurgery for Metastatic Brain Tumors from Malignant Melanomas: A Japanese Multi-Institutional Cooperative and Retrospective Cohort Study (JLGK1501).* Stereotactic and Functional Neurosurgery, 2018. **96**(3): p. 162-171.

9. Minniti, G., et al., *Outcomes of postoperative stereotactic radiosurgery to the resection cavity versus stereotactic radiosurgery alone for melanoma brain metastases.* J Neurooncol, 2017. **132**(3): p. 455-462.

10. Rauschenberg, R., et al., *Impact of radiation, systemic therapy and treatment sequencing on survival of patients with melanoma brain metastases.* European Journal of Cancer, 2019. **110**: p. 11-20.

11. Bhatia, A., et al., *MRI radiomic features are associated with survival in melanoma brain metastases treated with immune checkpoint inhibitors.* Neuro-Oncology, 2019. **21**(12): p. 1578-1586.

12. Gorka, E., et al., *Dabrafenib Therapy in 30 Patients with Melanoma Metastatic to the Brain: a Single-centre Controlled Retrospective Study in Hungary.* Pathology and Oncology Research, 2018. **24**(2): p. 401-406.

13. Iorgulescu, J.B., et al., *Improved risk-adjusted survival for melanoma brain metastases in the era of checkpoint blockade immunotherapies: Results from a national cohort.* Cancer Immunology Research, 2018. **6**(9): p. 1039-1045.

14. Ahmed, K.A., et al., *Clinical outcomes of melanoma brain metastases treated with stereotactic radiosurgery and anti-PD-1 therapy, anti-CTLA-4 therapy, BRAF/MEK inhibitors, BRAF inhibitor, or conventional chemotherapy.* Ann Oncol, 2016. **27**(12): p. 2288-2294.

15. De La Fuente, M., et al., *Whole-brain radiotherapy in patients with brain metastases from melanoma.* CNS Oncology, 2015. **3**(6): p. 401-406.

16. Drago, J.Z., et al., *Clinical experience with combination BRAF/MEK inhibitors for melanoma with brain metastases: A real-life multicenter study.* Melanoma Research, 2019. **29**(1): p. 65-69.

17. Frakes, J.M., et al., *Potential role for LINAC-based stereotactic radiosurgery for the treatment of 5 or more radioresistant melanoma brain metastases.* Journal of Neurosurgery, 2015. **123**(5): p. 1261-1267.

18. Geukes Foppen, M.H., et al., *Clinical and radiological response of BRAF inhibition and MEK inhibition in patients with brain metastases from BRAF-mutated melanoma.* Melanoma Res, 2018. **28**(2): p. 126-133.

19. Le Rhun, E., et al., *Response assessment and outcome of combining immunotherapy and radiosurgery for brain metastasis from malignant melanoma.* ESMO Open, 2020. **5**(4).

20. Knispel, S., et al., *Impact of a preceding radiotherapy on the outcome of immune checkpoint inhibition in metastatic melanoma: A multicenter retrospective cohort study of the DeCOG.* Journal for ImmunoTherapy of Cancer, 2020. **8**(1).

21. McHugh, F.A., et al., *Metastatic melanoma: Surgical treatment of brain metastases – Analysis of 110 patients.* Journal of Clinical Neuroscience, 2020. **73**: p. 144-149.

22. Minniti, G., et al., *Stereotactic radiosurgery combined with nivolumab or Ipilimumab for patients with melanoma brain metastases: Evaluation of brain control and toxicity.* Journal for ImmunoTherapy of Cancer, 2019. **7**(1).

23. Tetu, P., et al., *Impact of radiotherapy administered simultaneously with systemic treatment in patients with melanoma brain metastases within MelBase, a French multicentric prospective cohort.* European Journal of Cancer, 2019. **112**: p. 38-46.

24. Pomeranz Krummel, D.A., et al., *Impact of Sequencing Radiation Therapy and Immune Checkpoint Inhibitors in the Treatment of Melanoma Brain Metastases.* International Journal of Radiation Oncology Biology Physics, 2020. **108**(1): p. 157-163.

25. Rahman, R., et al., *The impact of timing of immunotherapy with cranial irradiation in melanoma patients with brain metastases: intracranial progression, survival and toxicity.* Journal of Neuro-Oncology, 2018. **138**(2): p. 299-306.

26. Schmidberger, H., et al., *Long-term survival of patients after ipilimumab and hypofractionated brain radiotherapy for brain metastases of malignant melanoma: sequence matters.* Strahlentherapie und Onkologie, 2018. **194**(12): p. 1144-1151.

27. Skrepnik, T., et al., *Improved time to disease progression in the brain in patients with melanoma brain metastases treated with concurrent delivery of radiosurgery and ipilimumab.* Oncoimmunology, 2017. **6**(3): p. e1283461.

28. Stera, S., et al., *Stereotactic radiosurgery combined with immune checkpoint inhibitors or kinase inhibitors for patients with multiple brain metastases of malignant melanoma.* Melanoma Res, 2019. **29**(2): p. 187-195.

29. Yusuf, M.B., et al., *Peri-SRS Administration of Immune Checkpoint Therapy for Melanoma Metastatic to the Brain: Investigating Efficacy and the Effects of Relative Treatment Timing on Lesion Response.* World Neurosurg, 2017. **100**: p. 632-640 e4.

30. Amaral, T., et al., *Immunotherapy plus surgery/radiosurgery is associated with favorable survival in patients with melanoma brain metastasis.* Immunotherapy, 2019. **11**(4): p. 297-309.

31. Gabani, P., et al., *Stereotactic radiosurgery and immunotherapy in melanoma brain metastases: Patterns of care and treatment outcomes.* Radiotherapy and Oncology, 2018. **128**(2): p. 266-273.

32. Kotecha, R., et al., *Melanoma brain metastasis: The impact of stereotactic radiosurgery, BRAF mutational status, and targeted and/or immune-based therapies on treatment outcome.* Journal of Neurosurgery, 2018. **129**(1): p. 50-59.

33. Martins, F., et al., *The combination of stereotactic radiosurgery with immune checkpoint inhibition or targeted therapy in melanoma patients with brain metastases: a retrospective study.* Journal of Neuro-Oncology, 2020. **146**(1): p. 181-193.

34. Sloot, S., et al., *Improved survival of patients with melanoma brain metastases in the era of targeted BRAF and immune checkpoint therapies.* Cancer, 2018. **124**(2): p. 297-305.

35. Wattson, D.A., et al., *Survival patterns following brain metastases for patients with melanoma in the MAP-kinase inhibitor era.* Journal of Neuro-Oncology, 2015. **123**(1): p. 75-84.

36. Stokes, W.A., et al., *Impact of immunotherapy among patients with melanoma brain metastases managed with radiotherapy.* Journal of Neuroimmunology, 2017. **313**: p. 118-122.

37. Tio, M., et al., *Survival and prognostic factors for patients with melanoma brain metastases in the era of modern systemic therapy.* Pigment Cell and Melanoma Research, 2018. **31**(4): p. 509-515.

38. Alvarez-Breckenridge, C., et al., *Upfront Surgical Resection of Melanoma Brain Metastases Provides a Bridge Toward Immunotherapy-Mediated Systemic Control.* Oncologist, 2019. **24**(5): p. 671-679.

39. Amaral, T., et al., *Combined immunotherapy with nivolumab and ipilimumab with and without local therapy in patients with melanoma brain metastasis: a DeCOG* study in 380 patients.* Journal for ImmunoTherapy of Cancer, 2020. **8**(1).

40. Cohen-Inbar, O., et al., *The effect of timing of stereotactic radiosurgery treatment of melanoma brain metastases treated with ipilimumab.* Journal of Neurosurgery, 2017. **127**(5): p. 1007-1014.

41. White, R.J., et al., *Melanoma brain metastases: is it time to eliminate radiotherapy?* Journal of Neuro-Oncology, 2020. **149**(1): p. 27-33.

42. Tio, M., et al., *Survival and prognostic factors for patients with melanoma brain metastases in the era of modern systemic therapy.* Pigment cell & melanoma research, 2018. **31**(4): p. 509-515.

43. Robin, T.P., et al., *Immune checkpoint inhibitors and radiosurgery for newly diagnosed melanoma brain metastases.* Journal of Neuro-Oncology, 2018. **140**(1): p. 55-62.

44. Davies, M.A., et al., *Dabrafenib plus trametinib in patients with BRAFV600-mutant melanoma brain metastases (COMBI-MB): a multicentre, multicohort, open-label, phase 2 trial.* The Lancet Oncology, 2017. **18**(7): p. 863-873.

45. Goldberg, S.B., et al., *Pembrolizumab for patients with melanoma or non-small-cell lung cancer and untreated brain metastases: early analysis of a non-randomised, open-label, phase 2 trial.* The Lancet Oncology, 2016. **17**(7): p. 976-983.

46. Gupta, A., et al., *RADVAN: A randomised phase 2 trial of WBRT plus vandetanib for melanoma brain metastases-results and lessons learnt.* British Journal of Cancer, 2016. **115**(10): p. 1193-1200.

47. Hauswald, H., et al., *Whole-brain helical tomotherapy with integrated boost for brain metastases in patients with malignant melanoma - final results of the BRAINRT trial.* Cancer Management and Research, 2019. **11**: p. 4669-4676.

48. Roche, H.-L., *A Study of Vemurafenib in Metastatic Melanoma Participants With Brain Metastases*. 2016: ClinicalTrials.gov.

49. Hong, A.M., et al., *Adjuvant Whole-Brain Radiation Therapy Compared With Observation After Local Treatment of Melanoma Brain Metastases: A Multicenter, Randomized Phase III Trial.* J Clin Oncol, 2019. **37**(33): p. 3132-3141.

50. Kluger, H.M., et al., *Long-Term Survival of Patients With Melanoma With Active Brain Metastases Treated With Pembrolizumab on a Phase II Trial.* J Clin Oncol, 2019. **37**(1): p. 52-60.

51. Long, G.V., et al., *Combination nivolumab and ipilimumab or nivolumab alone in melanoma brain metastases: a multicentre randomised phase 2 study.* The Lancet Oncology, 2018. **19**(5): p. 672-681.

52. McArthur, G.A., et al., *Vemurafenib in metastatic melanoma patients with brain metastases: An open-label, single-arm, phase 2, multicentre study.* Annals of Oncology, 2017. **28**(3): p. 634-641.

53. McQuade, J.L., et al., *A phase I study of TPI 287 in combination with temozolomide for patients with metastatic melanoma.* Melanoma Research, 2016. **26**(6): p. 604-608.

54. Tawbi, H.A., et al., *Combined Nivolumab and Ipilimumab in Melanoma Metastatic to the Brain.* N Engl J Med, 2018. **379**(8): p. 722-730.

55. Squibb, *A Multi-Center Phase II Study to Evaluate Tumor Response to Ipilimumab (BMS-734016) Monotherapy in Subjects With Melanoma Brain Metastases*. 2014: ClinicalTrials.gov.

56. Gummadi, T.Z., B. Y.; Valpione, S.; Kim, C.; Kottschade, L. A.; Mittapalli, R. K.; Chiarion-Sileni, V.; Pigozzo, J.; Elmquist, W. F.; Dudek, A. Z., *Impact of BRAF mutation and BRAF inhibition on melanoma brain metastases.* Melanoma Research, 2015. **25**(1): p. 75-79.

57. Partl, R.F., G.; Kaiser, J.; Kronhuber, E.; Cetin-Strohmer, K.; Steffal, C.; Böhmer-Breitfelder, B.; Mayer, J.; Avian, A.; Berghold, A., *KPS/LDH index: a simple tool for identifying patients with metastatic melanoma who are unlikely to benefit from palliative whole brain radiotherapy.* Supportive Care in Cancer, 2016. **24**(2): p. 523-528.

58. Chowdhury, I.H.O., E.; McMillan, M. T.; Miller, D.; Kolker, J. D.; Kurtz, G.; Dorsey, J. F.; Nagda, S. N.; Geiger, G. A.; Brem, S.; O'Rourke, D. M.; Zager, E. L.; Gangadhar, T.; Schuchter, L.; Lee, J. Y.; Alonso-Basanta, M., *Novel risk scores for survival and intracranial failure in patients treated with radiosurgery alone to melanoma brain metastases.* Radiation Oncology, 2015; . **10**: p. 248.

59. Bian, S.X.R., D.; Liu, J.; Yang, D.; Groshen, S.; Zada, G.; Trakul, N.; Wong, M. K.; Yu, C.; Chang, E. L., *Prognostic factors for melanoma brain metastases treated with stereotactic radiosurgery.* Journal of neurosurgery, 2016. **125**: p. 31-39.

60. Rodenburg, R.J.H., P. E.; Ho, V. K. Y.; Beerepoot, L. V., *Validation of the Chowdhury overall survival score in patients with melanoma brain metastasis treated with Gamma Knife Radiosurgery.* Journal of Neuro-Oncology, 2018. **138**(2): p. 391-399.

61. Ramos, R.I.B., M. A.; Wu, J.; Jones, P.; Chang, S. C.; Kiyohara, E.; Tran, K.; Zhang, X.; Stern, S. L.; Izraely, S.; Sagi-Assif, O.; Witz, I. P.; Davies, M. A.; Mills, G. B.; Kelly, D. F.; Irie, R. F.; Hoon, D. S. B., *Upregulation of cell surface GD3 ganglioside phenotype is associated with human melanoma brain metastasis.* Molecular Oncology, 2020. **14**(8): p. 1760-1778.

62. Gallaher, I.S.W., Y.; DeFor, T. E.; Dusenbery, K. E.; Lee, C. K.; Hunt, M. A.; Lin, H. Y.; Yuan, J., *BRAF mutation is associated with improved local control of melanoma brain metastases treated with Gamma Knife radiosurgery.* Frontiers in Oncology, 2016. **6**.

63. Sperduto, P.W.J., W.; Brown, P. D.; Braunstein, S.; Sneed, P.; Wattson, D. A.; Shih, H. A.; Bangdiwala, A.; Shanley, R.; Lockney, N. A.; Beal, K.; Lou, E.; Amatruda, T.; Sperduto, W. A.; Kirkpatrick, J. P.; Yeh, N.; Gaspar, L. E.; Molitoris, J. K.; Masucci, L.; Roberge, D.; Yu, J.; Chiang, V.; Mehta, M., *The Prognostic Value of BRAF, C-KIT, and NRAS Mutations in Melanoma Patients With Brain Metastases.* International Journal of Radiation Oncology Biology Physics, 2017. **98**(5): p. 1069-1077.

64. Xu, Z.L., C. C.; Ramesh, A.; Mueller, A. C.; Schlesinger, D.; Cohen-Inbar, O.; Shih, H. H.; Sheehan, J. P., *BRAF V600E mutation and BRAF kinase inhibitors in conjunction with stereotactic radiosurgery for intracranial melanoma metastases.* Journal of Neurosurgery, 2017. **126**(3): p. 726-734.

65. Schaule, J.S., J.; Kroeze, S. G. C.; Blanck, O.; Blanck, O.; Stera, S.; Kahl, K. H.; Roeder, F.; Combs, S. E.; Combs, S. E.; Combs, S. E.; Kaul, D.; Claes, A.; Schymalla, M. M.; Adebahr, S.; Adebahr, S.; Adebahr, S.; Eckert, F.; Lohaus, F.; Lohaus, F.; Lohaus, F.; Abbasi-Senger, N.; Henke, G.; Szuecs, M.; Geier, M.; Sundahl, N.; Buergy, D.; Dummer, R.; Guckenberger, M., *Predicting survival in melanoma patients treated with concurrent targeted- or immunotherapy and stereotactic radiotherapy.* Radiation Oncology, 2020. **15**(1).

66. Frinton, E., et al., *Metastatic melanoma: prognostic factors and survival in patients with brain metastases.* Journal of Neuro-Oncology, 2017. **135**(3): p. 507-512.

67. Mastorakos, P., et al., *BRAF V600 Mutation and BRAF Kinase Inhibitors in Conjunction With Stereotactic Radiosurgery for Intracranial Melanoma Metastases: A Multicenter Retrospective Study.* Neurosurgery, 2019. **84**(4): p. 868-880.

68. Zhang, M., et al., *Intracranial Tumor Control After Immune-Related Adverse Events and Discontinuation of Immunotherapy for Melanoma.* World Neurosurg, 2020. **144**.

69. Maxwell, R., et al., *BRAF-V600 mutational status affects recurrence patterns of melanoma brain metastasis.* Int J Cancer, 2017. **140**(12): p. 2716-2727.

70. Badakhshi, H.E., F.; Budach, V.; Ghadjar, P.; Zschaeck, S.; Kaul, D., *Are prognostic indices for brain metastases of melanoma still valid in the stereotactic era?* Radiation Oncology, 2018. **13**(1).

71. Zubatkina, I. and P. Ivanov, *Early imaging radioresponsiveness of melanoma brain metastases as a predictor of patient prognosis.* Journal of Neurosurgery, 2018. **129**(2): p. 354-365.

72. Ferrel, E.A.R., A. T.; Kaya, E. A.; Carlson, J. D.; Carlson, J. D.; Wagner, A.; Mackay, A. R.; Call, J. A.; Demakas, J. J.; Lamoreaux, W. T.; Fairbanks, R. K.; Cooke, B. S.; Peressini, B.; Lee, C. M., *Retrospective study of metastatic melanoma and renal cell carcinoma to the brain with multivariate analysis of prognostic pre-treatment clinical factors.* International Journal of Molecular Sciences, 2016. **17**(3).

73. Kavouridis, V.K.H., M.; Hulsbergen, A. F. C.; Lo, Y. T.; Reardon, D. A.; Aizer, A. A.; Iorgulescu, J. B.; Smith, T. R., *Survival and prognostic factors in surgically treated brain metastases.* Journal of Neuro-Oncology, 2019. **143**(2): p. 359-367.

74. Hirshman, B.R.W., B. R.; Ali, M. A.; Schupper, A. J.; Proudfoot, J. A.; Goetsch, S. J.; Carter, B. S.; Sinclair, G.; Bartek, J., Jr.; Chiang, V.; Fogarty, G.; Hong, A.; Chen, C. C., *Cumulative Intracranial Tumor Volume Augments the Prognostic Value of Diagnosis-Specific Graded Prognostic Assessment Model for Survival in Patients with Melanoma Cerebral Metastases.* Neurosurgery, 2018. **83**(2): p. 237-244.

75. Kano, H.M.-R., A.; Iyer, A.; Weiner, G. M.; Mousavi, S. H.; Kirkwood, J. M.; Tarhini, A. A.; Flickinger, J. C.; Dade Lunsford, L., *Comparison of prognostic indices in patients who undergo melanoma brain metastasis radiosurgery.* Journal of Neurosurgery, 2018. **128**(1): p. 14-22.

76. Bates, J.E.Y., P.; Usuki, K. Y.; Walter, K. A.; Huggins, C. F.; Okunieff, P.; Milano, M. T., *Brain metastasis from melanoma: the prognostic value of varying sites of extracranial disease.* Journal of Neuro-Oncology, 2015. **125**(2): p. 411-418.

77. Wilkins, A.F., A.; W Corbett, R.; Bloomfield, A.; Porta, N.; Morris, S.; Ali, Z.; Larkin, J.; Harrington, K., *The melanoma-specific graded prognostic assessment does not adequately discriminate prognosis in a modern population with brain metastases from malignant melanoma.* British Journal of Cancer, 2015. **113**(9): p. 1275-1281.

78. Rades, D.S., L.; Janssen, S.; Schild, S. E., *Prognostic factors after whole-brain radiotherapy alone for brain metastases from malignant melanoma.* Anticancer Research, 2016. **36**(12): p. 6637-6640.

79. Sehmisch, L., S. Schild, and D. Rades, *Development of a Survival Score for Patients with Cerebral Metastases from Melanoma.* Anticancer Research, 2017. **37**(1): p. 249-252.

80. Marzese, D.M., et al., *Brain metastasis is predetermined in early stages of cutaneous melanoma by CD44v6 expression through epigenetic regulation of the spliceosome.* Pigment Cell and Melanoma Research, 2015. **28**(1): p. 82-93.

81. Farris, M.M., E. R.; Cramer, C. K.; Hughes, R.; olph, D. M.; Ayala-Peacock, D. N.; Bourl; , J. D.; Ruiz, J.; Watabe, K.; Laxton, A. W.; Tatter, S. B.; Zhou, X.; Chan, M. D., *Brain Metastasis Velocity: A Novel Prognostic Metric Predictive of Overall Survival and Freedom From Whole-Brain Radiation Therapy After Distant Brain Failure Following Upfront Radiosurgery Alone.* International Journal of Radiation Oncology Biology Physics, 2017. **98**(1): p. 131-141.
